# Design and synthesis of a novel quinoline thiazolidinedione hybrid as a potential antidiabetic PPARγ modulator

**DOI:** 10.1038/s41598-025-03387-9

**Published:** 2025-06-01

**Authors:** Ayman M. Ibrahim, Mai E. Shoman, Radwa Taher Mohie el-dien, Entesar Ali Saber, Mahmoud Abdelnaser, Sherif A. Maher, Alaa M. Hayallah, Mahmoud Abdul-Aziz El-Rehany, Gamal El-Din A. Abuo-Rahma

**Affiliations:** 1 Department of Pharmaceutical Chemistry, Faculty of Pharmacy, Deraya University, New Minia, 61111 Egypt; 2 Department of Medicinal Chemistry, Faculty of Pharmacy, Minia University, Minia, 61519 Egypt; 3 Department of Pharmacognosy, Faculty of Pharmacy, New Valley University, El-kharga City, Egypt; 4 Department of Medical science, Histology and Cell Biology, Faculty of Pharmacy, Deraya University, New Minia, 61111 Egypt; 5 Department of Biochemistry, Faculty of Pharmacy, Deraya University, New Minia, 61111 Egypt; 6 Department of Biochemistry, Faculty of pharmacy, New Valley University, El-kharga City, Egypt; 7 Department of Pharmaceutical Organic Chemistry, Faculty of Pharmacy, Assiut University, Assiut, 71526 Egypt; 8 Pharmaceutical Chemistry Department, Faculty of Pharmacy, Sphinx University, New Assiut, Egypt

**Keywords:** Quinoline, Thiazolidinedione, PPARγ, Antidiabetic, SPPARMs, Biochemistry, Chemical biology, Drug discovery, Chemistry

## Abstract

Peroxisome proliferator-activated receptor γ (PPARγ) remains a critical target for antidiabetic drug development due to its role in glucose and lipid metabolism. However, the adverse effects associated with full agonists of the thiazolidinedione (TZD) class, such as weight gain and hepatotoxicity, limit their clinical utility. Herein, we report the design and synthesis of (*Z*)-5-benzylidene-3-((2-chloroquinolin-3-yl)methyl)thiazolidine-2,4-dione (compound **7**), a novel TZD derivative that functions as a potential PPARγ modulator. Compound **7** reduced blood glucose level (BGL) by 22.33% after 15 days of treatment with a daily single oral dose, demonstrating an antidiabetic effect comparable to TZDs. Additionally, it elevated PPARγ expression to 75% of the activation level induced by Pioglitazone (PIO). Further characterization of its safety profile reveals that compound **7** is safer on the liver compared to PIO, as alanine transaminase (ALT) and aspartate transaminase (AST) levels remained significantly lower (147.4 ± 4.2 IU/L and 229.9 ± 2.7 IU/L, respectively). Moreover, compound **7** exerts a protective effect on hepatic and pancreatic tissues. Computational metabolic studies predict that compound **7** does not produce toxic metabolites or undergo hydrolysis of the TZD ring, contributing to its improved safety. The docking of Compound **7** into the PPARγ ligand-binding domain (LBD) demonstrates a unique binding mode, positioning it centrally within the LBD and interacting with key amino acids critical for selective modulation. These findings emphasize the potential of compound **7** as a selective PPARγ modulator to dissociate insulin-sensitizing effects from adverse side effects, offering a safer alternative to current TZD-based therapies.

**Supplementary Information:**

The online version contains supplementary material available at 10.1038/s41598-025-03387-9.

## Introduction

**Fig. 1:**
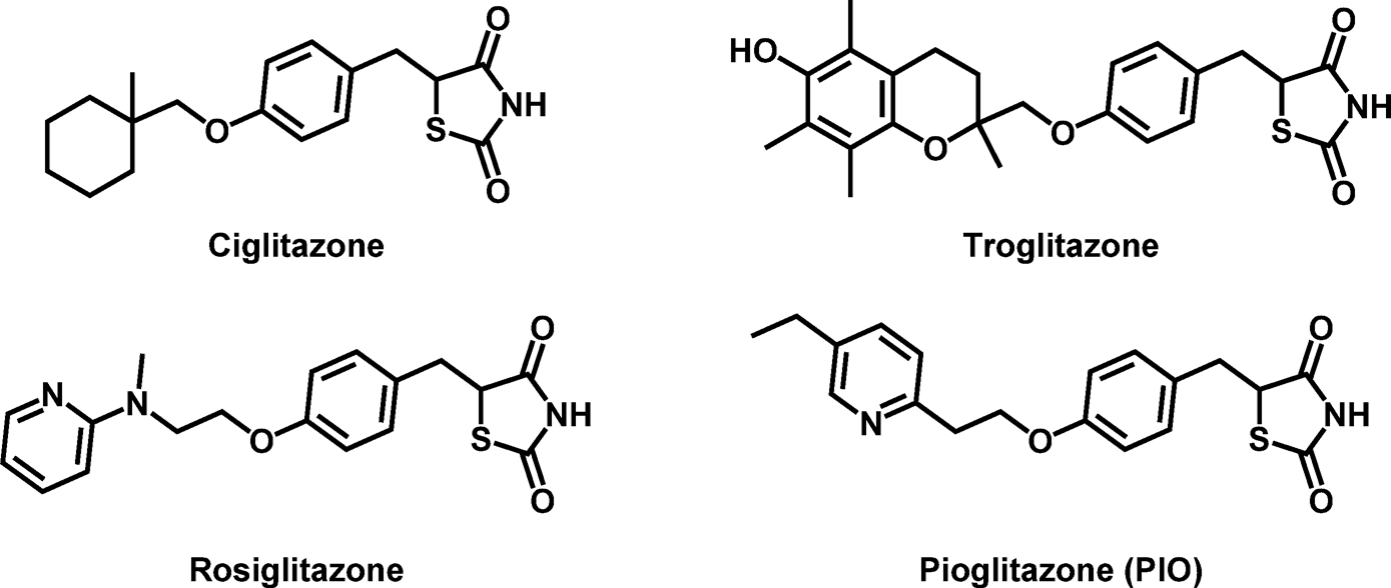
Chemical structures of glitazones.

Type II diabetes mellitus (T2DM) stands as a chronic metabolic disorder characterized by insulin resistance and impaired glucose homeostasis^1^ The therapeutic management of T2DM involves various drug classes, each targeting distinct aspects of glucose regulation. Among them, glitazones (Fig. 1), or thiazolidinediones (TZDs), have emerged as pharmacological agents that modulate insulin sensitivity and improve glycemic control^2^.

TZDs effectively lower hemoglobin A1c by approximately 1% as monotherapy in T2DM without causing hypoglycemia, unlike insulin or insulin secretagogues such as sulfonylureas; this makes them suitable for combination therapy with other antidiabetic agents^3^ Studies have shown that TZDs improved insulin sensitivity, where the randomized controlled trial has demonstrated that Rosiglitazone is more durable glycemic control compared to metformin or sulfonylurea^4^ Current practice guidelines approved PIO for biopsy-proven nonalcoholic steatohepatitis (NASH), while guidelines recommend TZDs to decrease androgen levels, enhance ovulation, and improve glucose tolerance in women with polycystic ovarian syndrome (PCOS), though not for first-line use in treating hirsutism or infertility^5^.

**Fig. 2:**
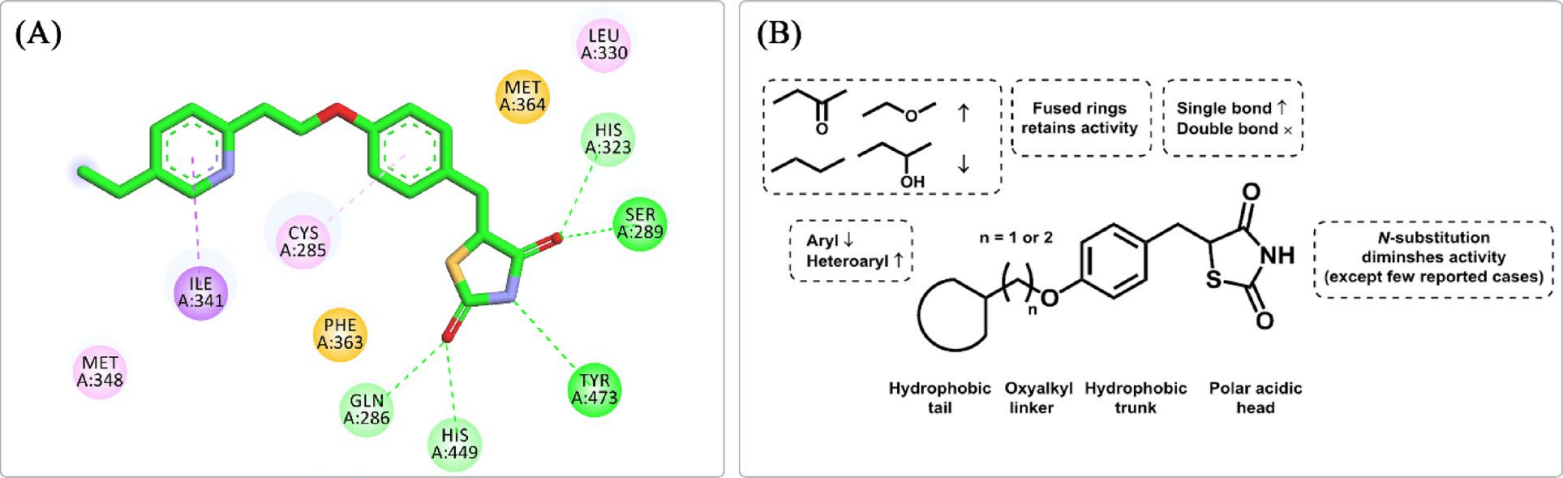
(**A**) Binding mode of PIO with key residues of PPARγ LBD. (**B**) Structure-activity relationship (SAR) of TZDs as PPARγ full agonists.

The primary molecular target of glitazones in antidiabetic action is the PPARγ^6^ PPARγ is a nuclear receptor that plays a crucial role in regulating glucose and lipid metabolism in various tissues, including adipose tissue, skeletal muscles, and liver^7,8^ Upon activation by TZDs, PPARγ forms a heterodimer with Retinoid X Receptor (RXR) and binds to specific PPAR response elements (PPREs) in the promoter region of target genes^9^ This activation results in the transcriptional regulation of pathways involved in insulin sensitivity, adipogenesis, and lipid metabolism, leading to improved glucose homeostasis^10,11^ TZDs are full agonists of PPARγ that form hydrogen bonds between their TZD head group and the ligand-binding domain (LBD) of PPARγ, specifically with the side chains of key residues His323, His449, and Tyr473 (Fig. 2). These interactions play a crucial role in stabilizing helix 12 (H12) in the active conformation leading to the formation of the activation function 2 (AF2) surface, which is necessary for full receptor activation^12^.

Despite their efficacy, glitazones are associated with a range of adverse effects, including weight gain and fluid retention. Long-term use of PIO raises concerns about cardiovascular safety, particularly an increased risk of heart failure and myocardial infarction^13^ PIO may elevate the risk of bone fractures and has been linked to cases of liver toxicity. Moreover, long-term use of PIO has also been linked to an increased risk of bladder cancer, raising concerns about safety profile^14,15^ Three TZDs received FDA approval: Troglitazone, Rosiglitazone, and PIO. Troglitazone was introduced in 1997 but withdrawn in 2000 due to liver toxicity concerns. Rosiglitazone and Pioglitazone were both approved in 1999, but Rosiglitazone faced restrictions due to cardiovascular concerns that were later lifted^5^.

**Fig. 3:**
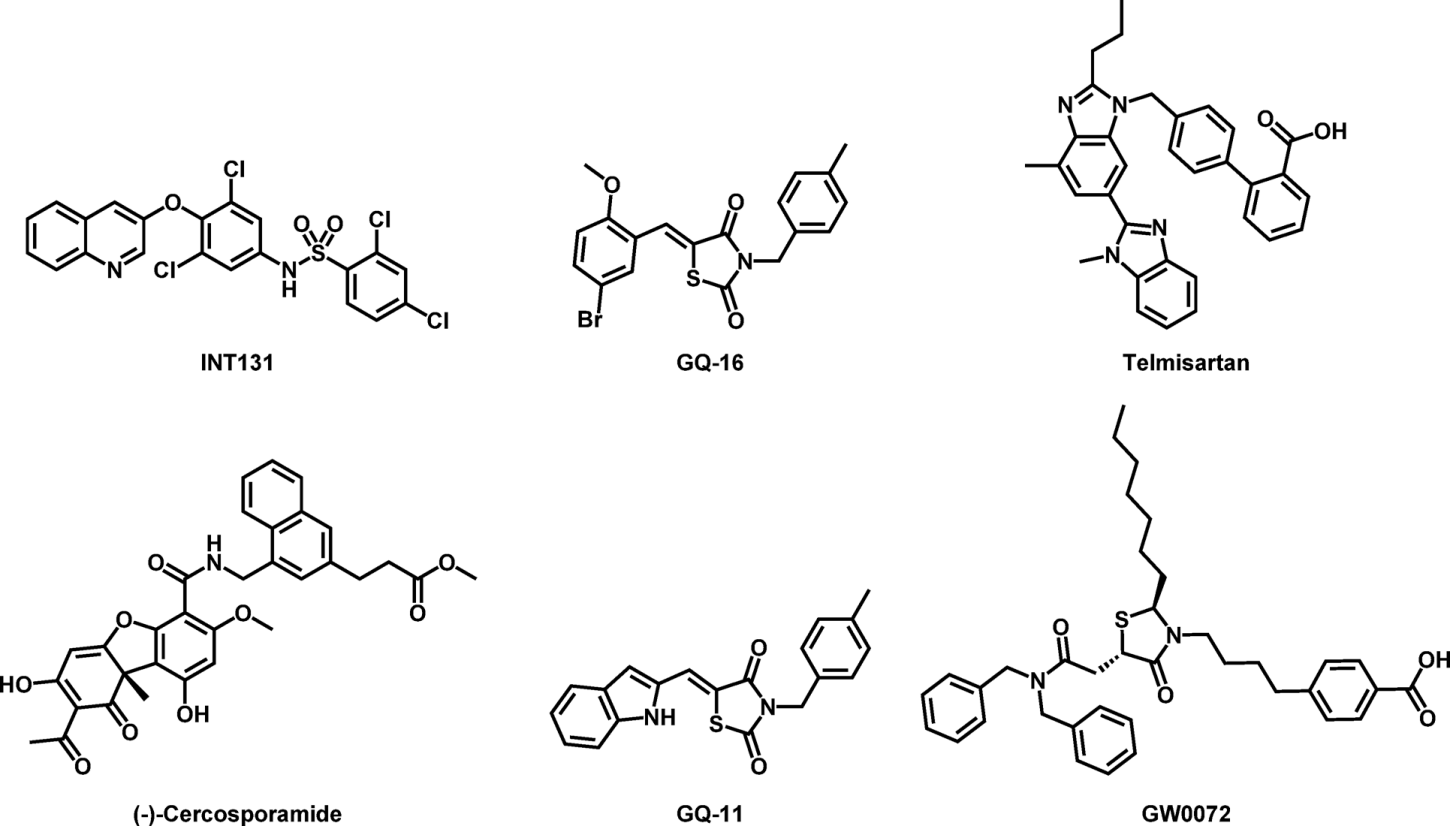
Chemical structures of SPPARMs.

Thus, a new prospective class of compounds appeared for the treatment of diabetes as selective PPARγ modulators (SPPARMs) (Fig. 3). These compounds have been demonstrated to retain favorable insulin-sensitizing effects while exhibiting little to no adverse effects. SPPARMs induce selective receptor conformations, engaging distinct signaling pathways, which leads to a more targeted and balanced activation of PPARγ^16^ This selective modulation enhances insulin sensitivity while minimizing common side effects such as weight gain, fluid retention, and cardiovascular risks, making them a safer alternative to traditional TZDs^17^.

**Fig. 4:**
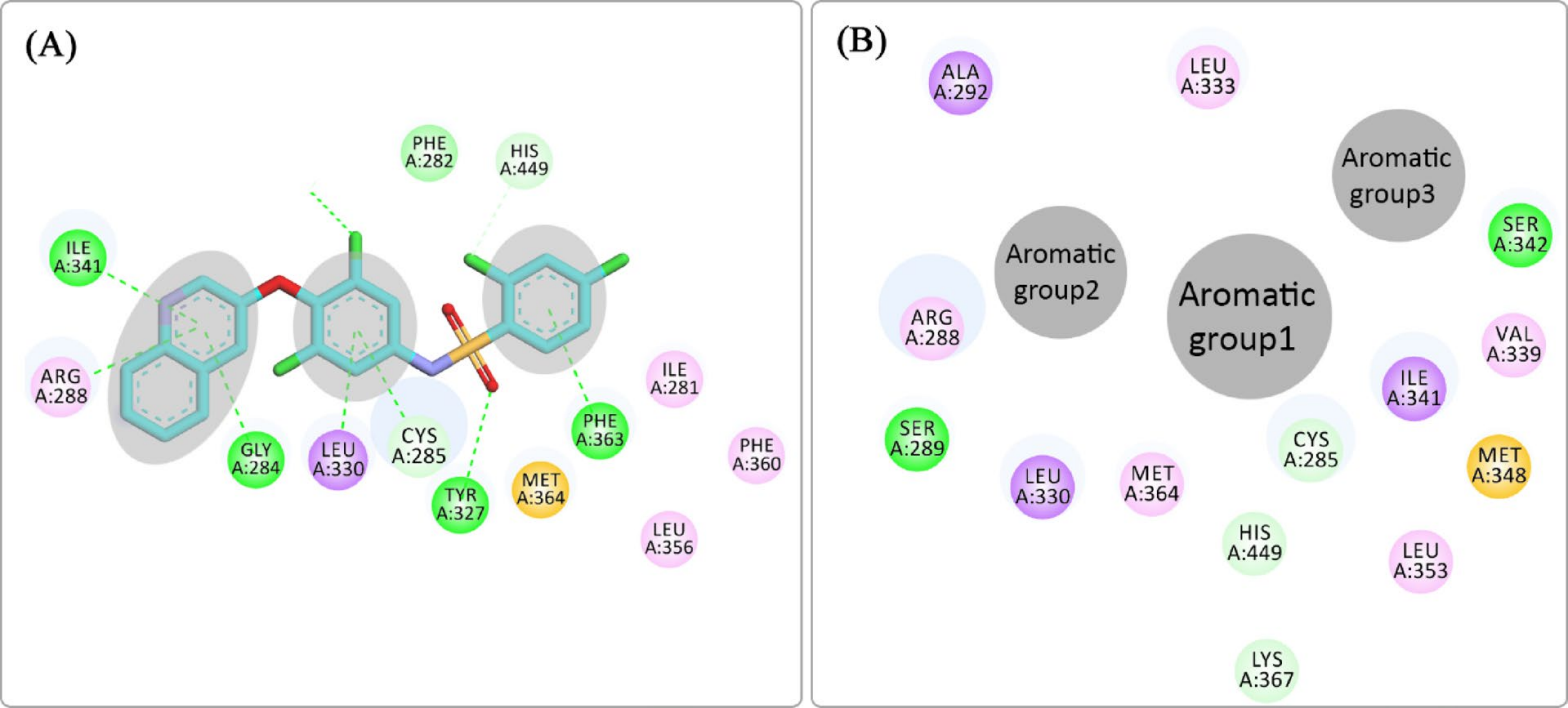
(**A**) Binding mode of **INT131** with key residues of PPAR LBDγ. (**B**) General structure features of SPPARMs.

SPPARMs, as shown in Fig. 3, do not share a general structure. However, they exhibit common binding features within the PPARγ LBD (Fig. 4). For example, **INT131**, a well-characterized SPPARM, binds to the LBD in a manner representative of this class^18^ Like other SPPARMs, **INT131** interacts hydrophobically with Cys285 of H3, a critical residue for partial activation, and engages Arg288 *via* hydrophobic/van der Waals forces (Fig. 4A). These interactions are often mediated by aromatic groups present within the SPPARM structure, as shown in Fig. 4B. This contrasts with full agonists like PIO, which stabilize the AF2 domain through strong electrostatic interactions with His323, Tyr473, and His449. The distinct binding mode of **INT131** (Fig. 4A) exemplifies the SPPARM signature: retention of key interactions with Cys285 and Arg288, coupled with the absence of direct stabilization of the AF2 domain. These features collectively contribute to the unique partial activation profile and reduced adverse effects associated with SPPARMs.

Therefore, motivated by the advantages of the SPPARMs, a novel derivative of the TZD scaffold has been designed with improved pharmacological properties. The design of this compound was based on several considerations. The investigations showed that toxicity of TZDs is most likely due to the hydrolysis of the TZD ring leading to formation of reactive intermediates, and hence TZD toxicity^19^ Additionally, it was observed that substitution at the nitrogen atom of the TZD ring in **GQ-11** prevents the cleavage of the TZD moiety, rendering it non-toxic to the liver^20^ Based on these observations, the first consideration was to modify the TZD ring in the desired compound by introducing a substitution at the nitrogen atom.

The other consideration is to enhance the designed compound’s binding with its target. A quinoline ring was selected as the nitrogen substituent. The aromatic nature of quinoline, characterized by its π-electron system, enables π-π stacking interactions with aromatic residues in the binding sites of biomolecules. Within the LBD of PPARγ, these interactions possible to occur with aromatic residues, including Phe282, Phe363, and His449. Additionally, the nitrogen atom within the quinoline ring contributes to hydrogen bonding, enhancing specificity and affinity for target binding sites^21^.

**Fig. 5:**
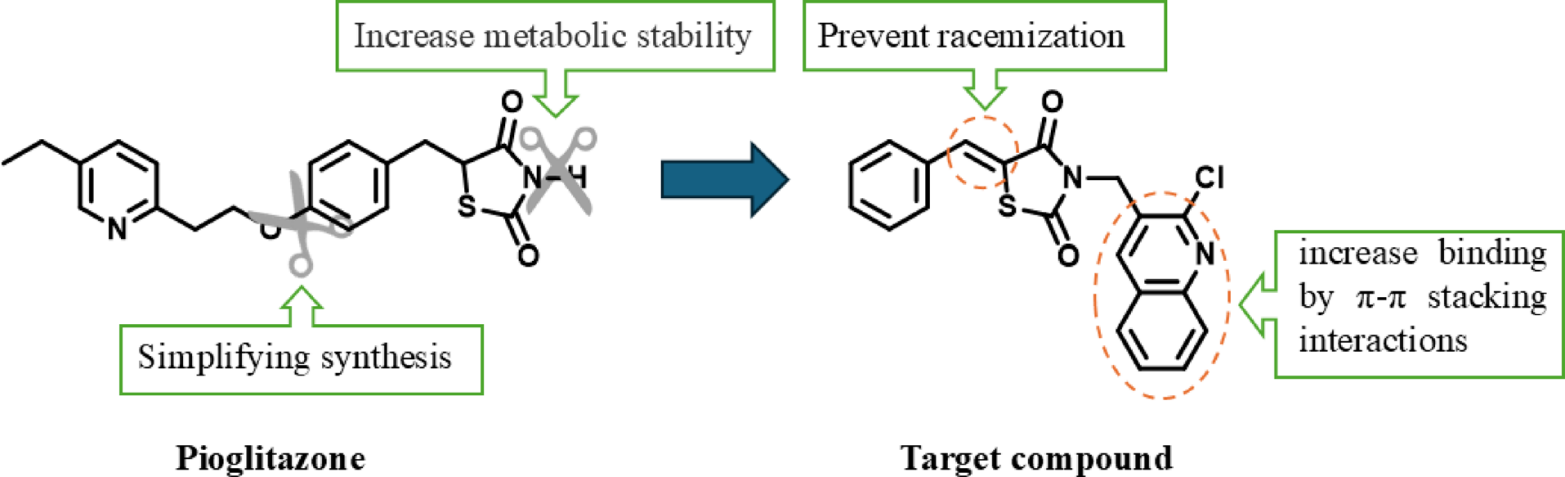
The design of the target molecule.

The final point depends on the selection of 5-benzylidene-2,4-thiazolidinedione moiety over 5-benzyl counterpart. The presence of the exocyclic double bond eliminates concerns regarding stereochemical instability and potential racemization. This structural preference facilitates access to the target compound without complexities associated with reduction methods of double bond as non-selectivity over other functional groups, toxicity, catalyst poisoning, and low to moderate yield. Combining these structural features, compound **7** was developed to achieve an optimized balance of safety, efficacy, and drug-like properties (Fig. 5)^22^.

## Results and discussion

### Chemistry

**Fig. 6:**
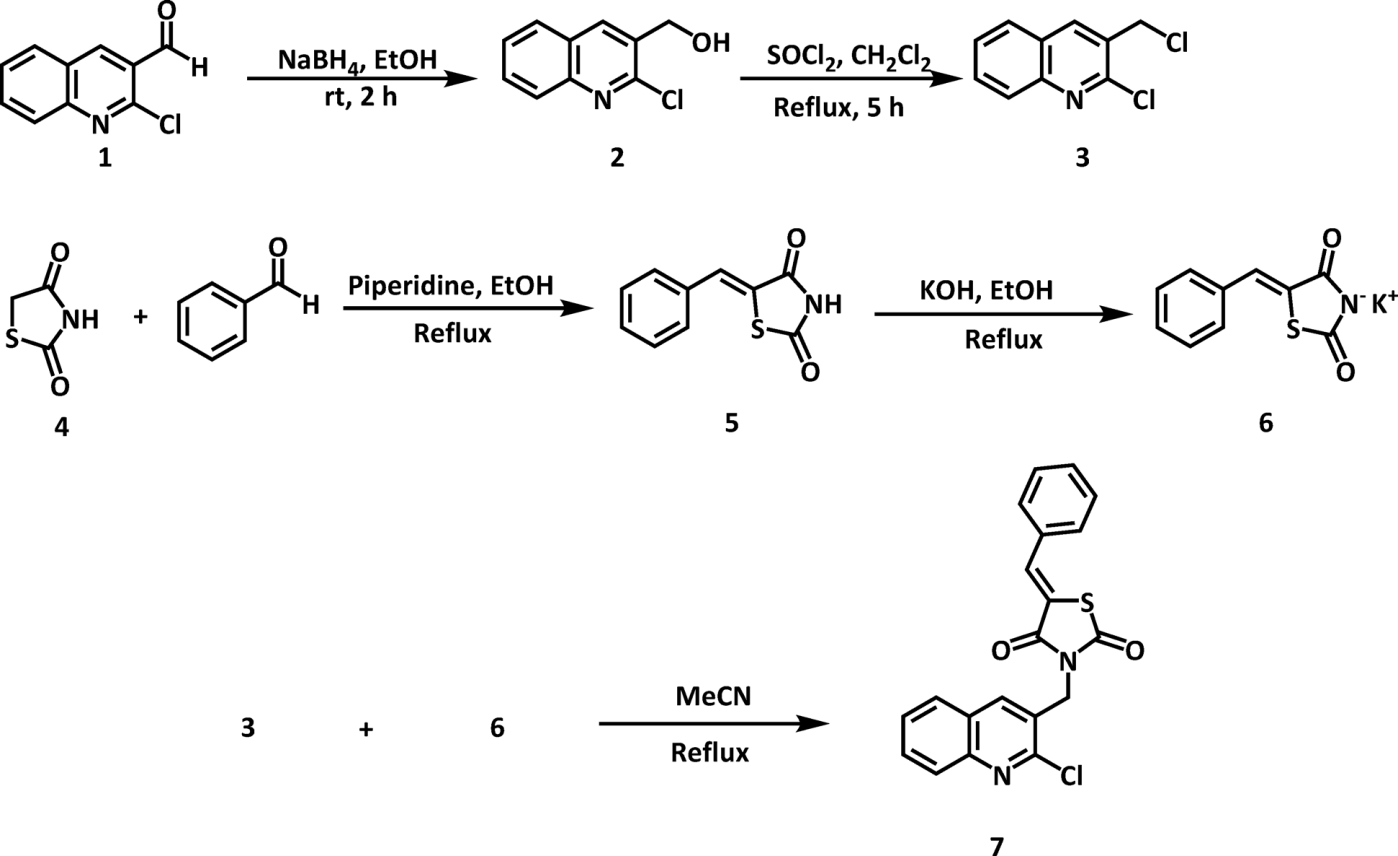
Synthetic pathway of target compound **7**.

The titled compound **7** was synthesized as per the route outlined in Fig. 6. Acetanilide was treated with Vilsmeier–Haack reagent (POCl_3_ and *N*,*N*-dimethyl formamide (DMF)) to form intermediate **1**. Intermediate **1** was then treated with solid NaBH_4_ in ethanol to afford 2-chloro-3-hydroxymethylquinoline **2**, followed by chlorination with SOCl_2_ in dichloromethane to obtain intermediate **3**. Heating intermediate **3** in acetonitrile under reflux with the potassium salt of (*Z*)-5-benzylidenethiazolidine-2,4-dione **6** formed the target compound **7**. The structure of TZD derivatives **7** was confirmed by^1^H and^13^C NMR. For instance, the^1^H NMR spectrum of compound **7** was characterized by a singlet signal assigned to the methylene protons (CH_2_) which experienced a downfield shift from δ 4.82 to 5.00 ppm (Figure S1). Notably, a characteristic signal for the methylene group appeared at δ 42.60 ppm. Moreover, the chemical shift of the methine proton shifted from δ 7.79 to 7.97 ppm. Also, the spectrum included a singlet at δ 8.40 ppm, corresponding to the H4 of the quinoline moiety. Another signal at δ 8.03 ppm represents the proton H5 of the quinoline nucleus. Additionally, a signal at δ 7.94 ppm corresponds to H8 of the quinoline moiety. The spectrum also exhibited a triplet at δ 7.79 ppm, representing H7 of the quinoline group. Furthermore, multiple resonances were observed for the phenyl group, including a multiplet at the range δ 7.54–7.64 ppm. DEPTQ-135 ^13^C NMR spectrum of compound 8a displayed 18 distinct signals (Figure S2). Among these signals, 9 signals were observed exhibiting a phase shift of 180° compared to the other 9 signals arising from CH and CH_3_ carbons. Notably, a characteristic signal originating from the methylene group appeared at δ 42.60 ppm. The peaks observed at 167.41 and 165.50 ppm were attributed to the two carbonyl groups present in the TZD ring. The molecular structure was further confirmed by mass spectrometry (ESI-MS, water), showing the molecular ion peak [M + H] + at m/z 380.5, consistent with the calculated molecular mass (Figure S3). The peak at *m/z* 434 could potentially be attributed to an adduct with the solvent, specifically [M + Na + MeOH]+. Crucially, the expected chlorine isotope peak at *m/z* 382.5 was also observed, with an intensity of approximately 32% relative to the [M + H] + 380.5 peak, confirming the presence of the chlorine atom in the molecule. HPLC analysis of compound **7** revealed a purity level of 95% (Figure S4). The synthesis of compound **7** is notably simpler and more straightforward, with a higher yield compared to the synthesis of PIO^23,24^ It involves fewer steps and relies on inexpensive and commercially available starting materials, making it a cost-effective and practical approach for large-scale production.

### Biological investigation

#### In vivo antidiabetic activity

**Fig. 7:**
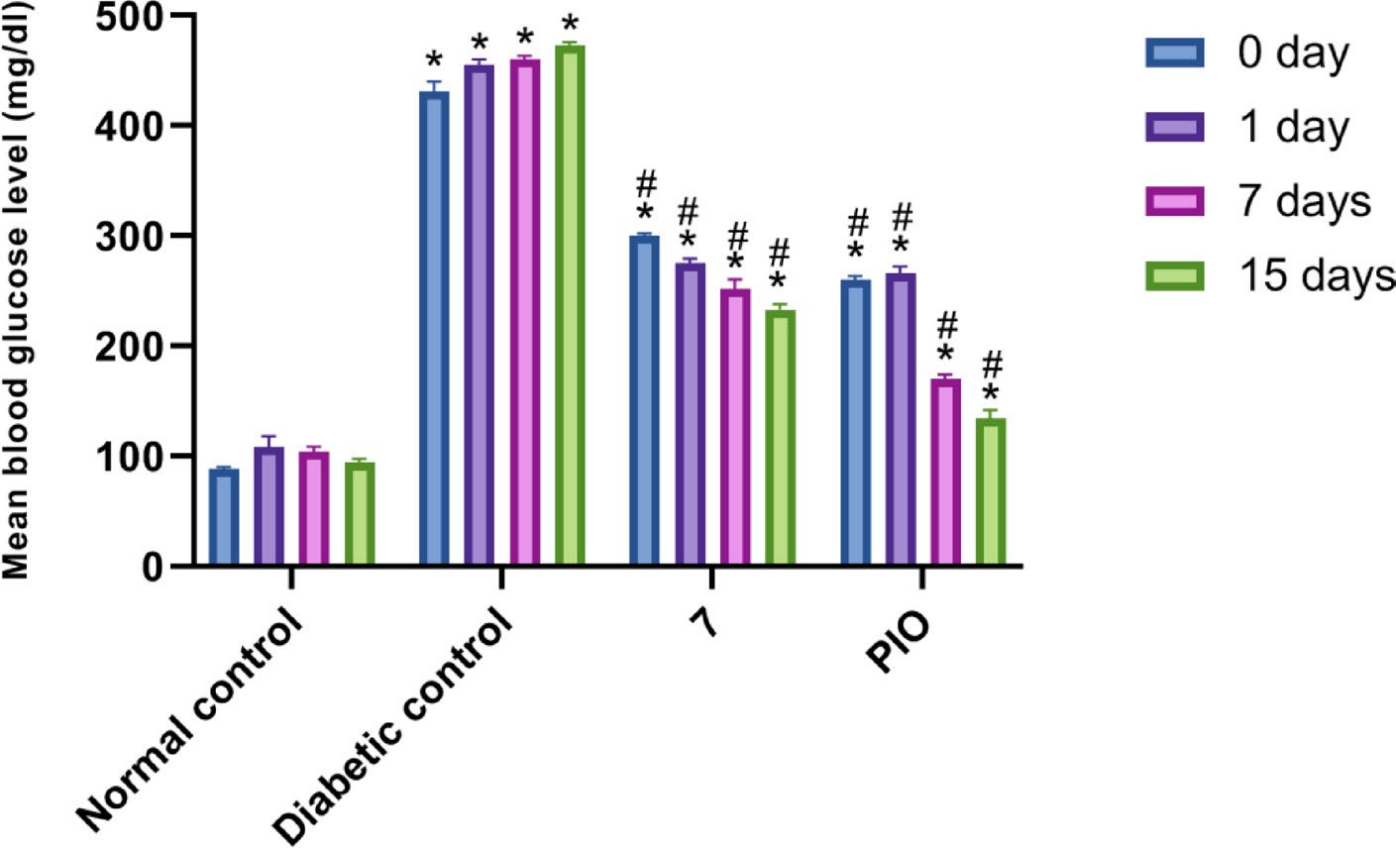
Antidiabetic activity of compound **7** in alloxan-induced diabetic rats. Data are analyzed by one-way ANOVA followed by Bonferroni *t*-test and expressed as mean ± SD from five observations; *p < 0.05 *versus* normal control; and #*p* < 0.05 *versus* diabetic control.

Compound **7** was evaluated on alloxan-induced diabetic rats for its blood glucose lowering effect. Besides control and diabetic groups, PIO was used as a reference drug with a single oral dose (36 mg/kg) to group III^25^ Compound **7** was also orally administered as an equimolar (1 mmole) single dose (38 mg/kg) to group IV. PIO and **7** were given in the form of a 0.25% carboxymethylcellulose (CMC) suspension and the fasting BGLs were monitored as per standard protocols on the 0-day, 1st, 7th, and 15th day of the commencement of the experiment^25,26^ The results are outlined in Fig. 7.

The data analysis showed that compound **7** and PIO effectively reduced blood glucose levels. On the 15th day, compound **7** demonstrated a decrease in BGL from 300 ± 2.23 mg/dL to 233 ± 5.05 mg/dL, while PIO exhibited a BGL reduction from 260 ± 3.5 mg/dL to 134 ± 7.74 mg/dL, in comparison to the diabetic group. Thus, compound **7** showed a promising blood glucose lowering effect with 22.33% along with the reference drug, PIO (48.46%).

#### PPARγ gene expression study

**Fig. 8:**
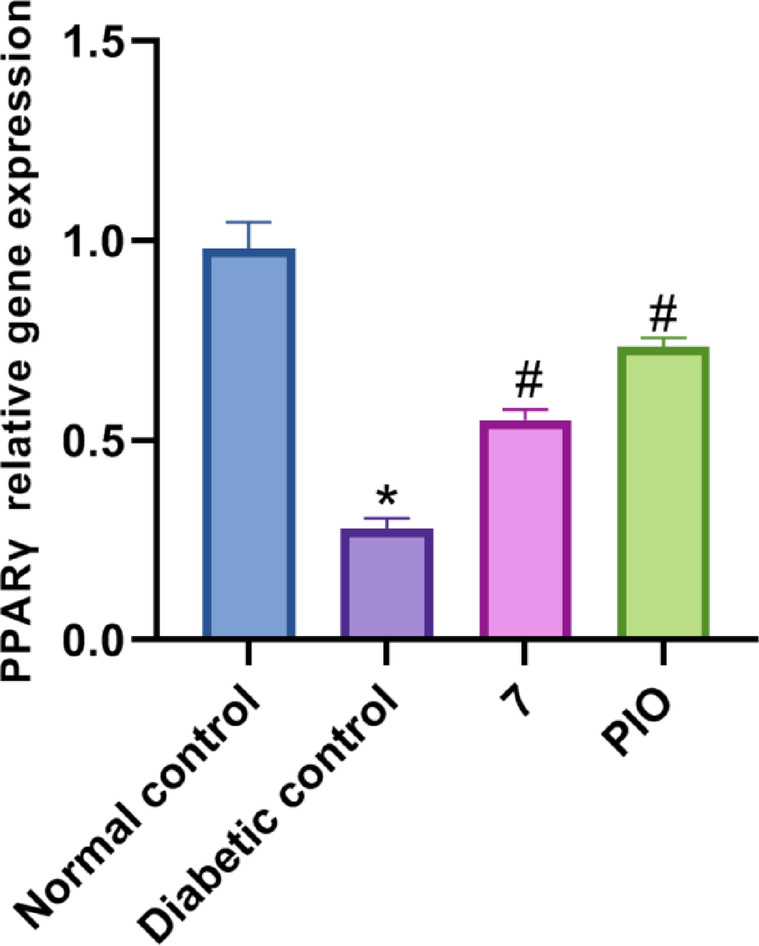
Effect of compound **7** on pancreatic PPARγ mRNA levels. Data are analyzed by one-way ANOVA followed by Bonferroni *t*-test and expressed as mean ± SD from five observations; *p < 0.05 *versus* normal control; and #*p* < 0.05 *versus* diabetic control.

PPAR**γ** gene expression analysis was done to evaluate the impact of compound **7** on modulating PPARγ gene. As shown in Fig. 8, diabetes significantly downregulated the expression of PPARγ gene, compared to the normal control group. However, in both **7**-treated group and PIO-treated group, the expression of PPARγ gene was markedly elevated, in contrast to the diabetic group. Thus, the increase in gene expression exerted by compound **7** supports its blood glucose lowering effect and its potential PPARγ activation. The transcriptional activity induced by SPPARMs is lower than that of full agonists. For instance, some partial agonists show transcriptional outputs ranging from 20 to 80% of full agonists like Rosiglitazone in reporter assays^27^ This partial activation modulates a subset of PPARγ-regulated genes, focusing on those involved in insulin sensitization while minimizing the activation of genes linked to adverse effects.

#### Body weight gain study

**Fig. 9:**
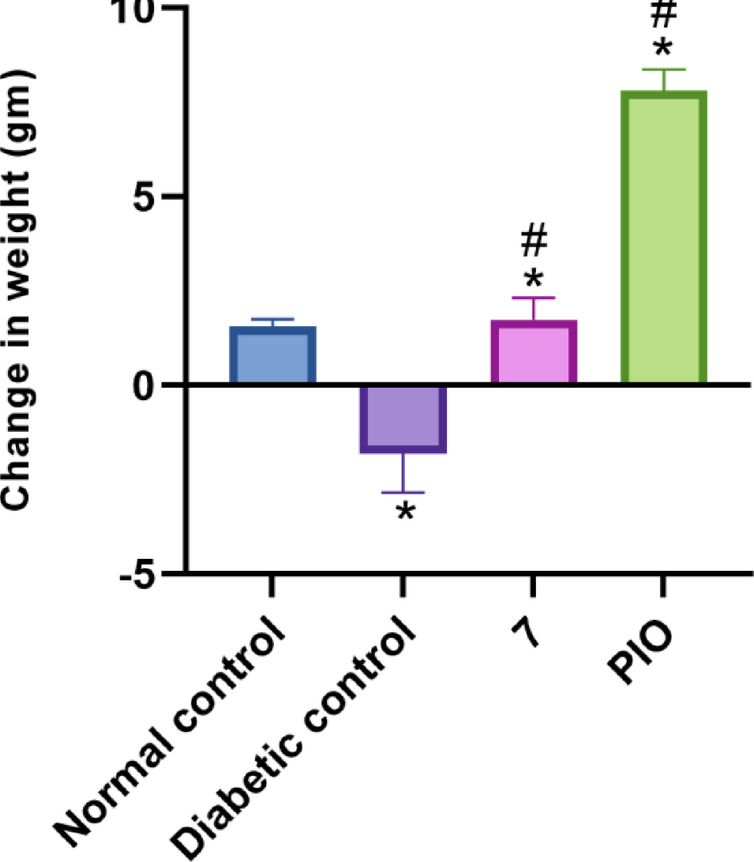
Effect of compound **7** on body weight gain in rats. Data are analyzed by one-way ANOVA followed by Bonferroni *t*-test and expressed as mean ± SD from five observations; *p < 0.05 *versus* normal control; and #*p* < 0.05 *versus* diabetic control.

Due to the earlier association of PPARγ full agonists with weight gain among treated animals, compound **7** was further analyzed for body weight gain (Fig. 9). In the diabetic control group, the body weight was decreased which may be attributed to the underlying diabetic condition. Administration of compound **7** resulted in a slight increase in body weight indicating that compound **7** has no significant effect on body weight compared to both the normal and diabetic control groups. In comparison, treatment with PIO induced a significant weight gain, which was notably higher than the changes observed with compound **7** and the normal control group.

#### Hepatotoxicity studies

**Fig. 10:**
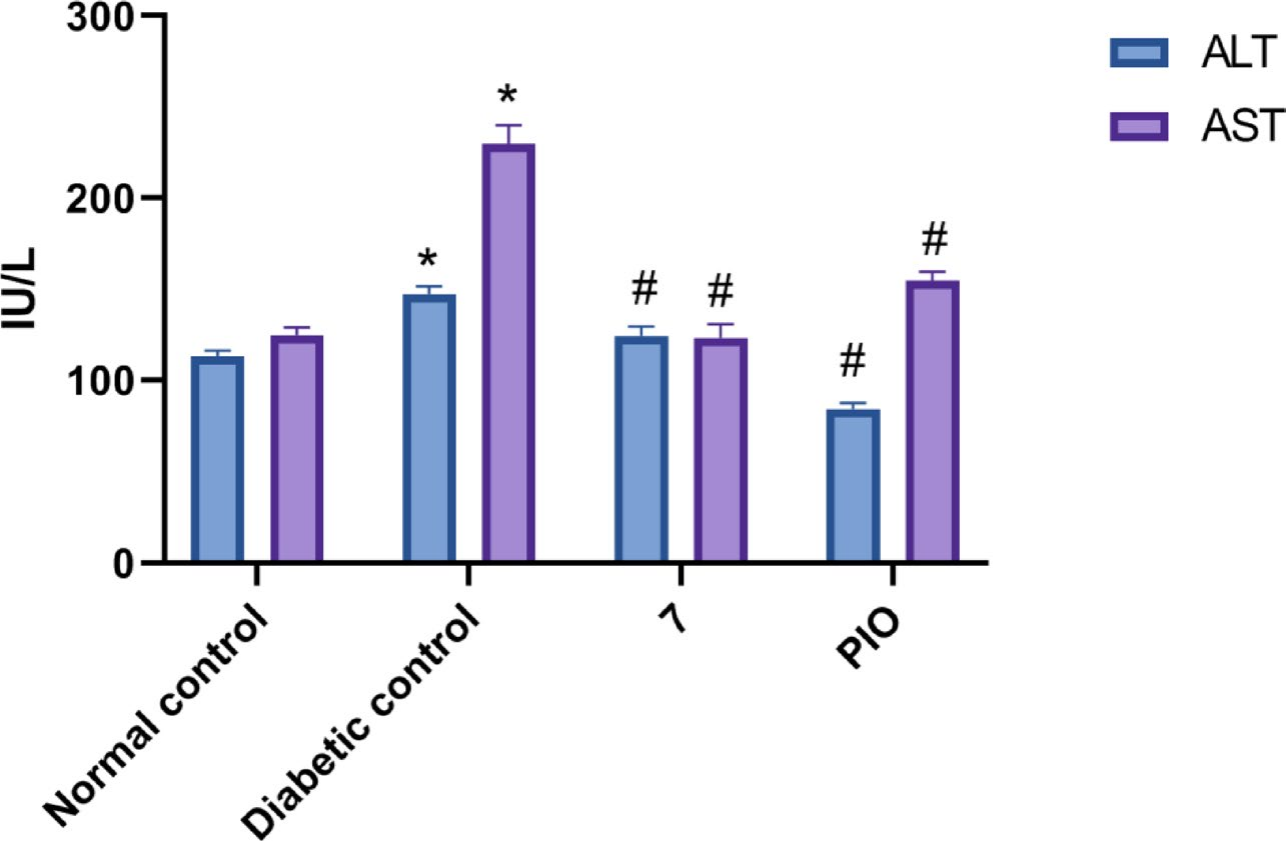
Effect of compound **7** on serum AST and ALT levels. Data are analyzed by one-way ANOVA followed by Bonferroni *t*-test and expressed as mean ± SD from five observations; *p < 0.05 *versus* normal control; and #*p* < 0.05 *versus* diabetic control.

Compound **7** was further analyzed for an increase or decrease in the levels of ALT and AST (Fig. 10). Normal control group showed ALT and AST levels of 113.2 ± 3.2 and 124.8 ± 4.4 IU/L, respectively. The diabetic control group served as a model for evaluating the impact of diabetes on liver function. The levels of ALT and AST were significantly elevated to 147.4 ± 4.2 IU/L and 229.9 ± 2.7 IU/L, respectively. This increase indicates impaired liver function associated with diabetes. Compound **7** was effective in bringing down the levels of ALT and AST to the normal range, with 124.3 ± 5.3 IU/L and 123.3 ± 7.6 IU/L, respectively. Treatment with PIO resulted in an elevated level of AST (154.7 ± 4.8 IU/L) when compared to the normal control group.

**Fig. 11:**
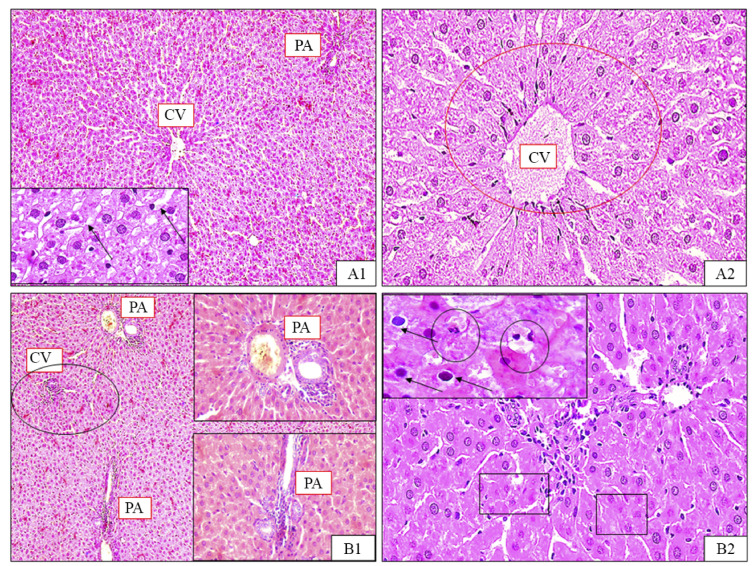
Photomicrographs of rat liver tissue for normal group (A1 & A2), and diabetic group (B1 & B2). (**A1**) Normal liver architecture with central veins (CV) and blood sinusoids in-between rows of hepatocytes (arrows). (**A2**) Hepatocytes are large polyhedral cells with vesicular nuclei and vacuolated and acidophilic cytoplasm. (**B1**) marked congestion of CV, vessels of the portal areas (PA) and blood sinusoids. Widely distributed patchy inflammation and necrosis mainly concentrated around the central veins (empty circle); (**B2**) Hepatic cord disorganization and dissolution. Varying degrees of cytoplasmic vacuolation (empty rectangles) and some nuclei showing pyknosis (arrows).

Histopathological examination of liver sections from the normal control group demonstrated normal hepatic architecture with characteristic hexagonal lobules. The tissue showed well-organized hepatic cords radiating from central veins, with intact portal triads at the periphery. Hepatocytes exhibited normal polyhedral morphology with vesicular nuclei and acidophilic cytoplasm, separated by clear sinusoidal spaces (Fig. 11A1, A2).

Liver sections from the diabetic control group exhibited severe disruption of hepatic architecture with disorganized hepatic cords and prominent sinusoidal dilation. Hepatocytes displayed extensive degenerative changes, including cytoplasmic vacuolation and nuclear abnormalities (pyknosis, fragmentation, and karyolysis). Dilated sinusoids, steatosis, glycogen depletion, and enlarged Kupffer cells were present. Notable features included marked central vein congestion, patchy necrosis, and perivenular inflammatory cell infiltration extending toward portal areas. These pathological changes indicate the severity of diabetes-induced hepatic injury (Fig. 11B1, B2).

**Fig. 12:**
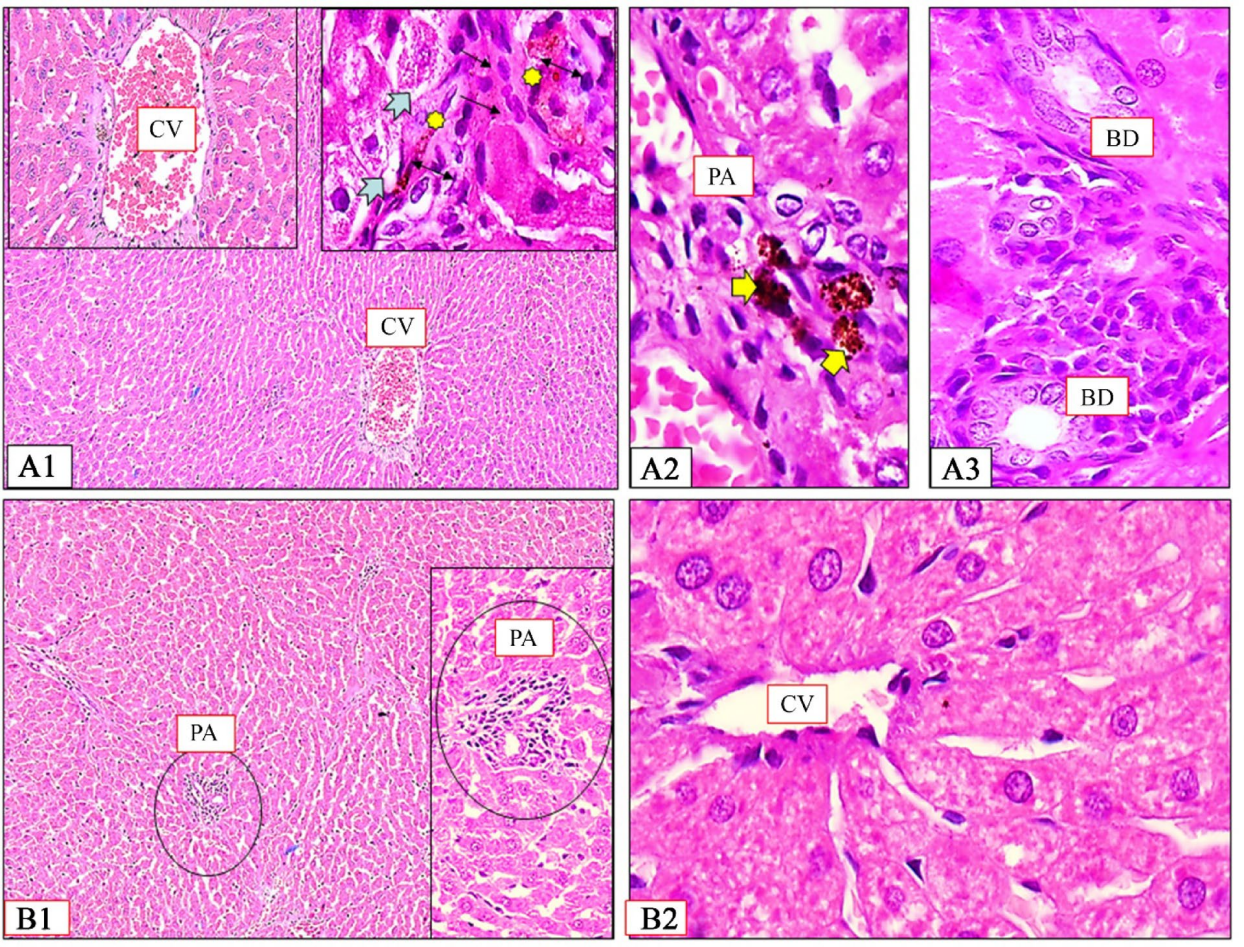
Photomicrographs of rat liver tissue of **7**-treated group (A1-A3) and PIO-treated group (B1 & B2). (**A1**) Hepatocytes have normal appearance but few hepatocytes appeared slightly vacuolated (tailed arrows). (**A2**) Kupffer cells appear inserted in the endothelial lining of hepatic sinusoids detected by particles of brown containing pigments (double arrows). (**A3**) Branching bile canaliculi (BC) seen in portal tracts. (**B1**) Classic hepatic lobules were roughly hexagonal in shape with CV forming their central axis from which cords of hepatocytes radiating like sun rays and surrounded at each corner by PA. (**B2**) Delicate collagen fibers in the interlobular septa and surrounding the liver cells and the blood sinusoids.

Treatment with compound **7** demonstrated notable amelioration of diabetes-induced hepatic changes. The liver sections showed improvement in hepatic architecture, with partially restored hexagonal lobular structure and organized central veins. Hepatocytes exhibited improved cellular integrity, radiating in well-defined cords from the central axis. The hepatic tissue displayed reduced inflammatory infiltration compared to the diabetic control group, with only mild collagen deposition in the interlobular septa and around sinusoids, indicating attenuation of diabetes-induced liver damage (Fig. 12A1-A3).

PIO treatment demonstrated moderate amelioration of alloxan-induced hepatic changes. The liver sections showed partial restoration of hepatic architecture, with hepatocytes arranged in radiating cords and relatively normal portal areas. While most hepatocytes displayed normal acidophilic cytoplasm and vesicular nuclei, scattered cells exhibited mild vacuolation. The tissue showed reduced inflammatory infiltration compared to diabetic controls, with minimal fibrous tissue surrounding hepatic lobules, central veins, and portal areas. Notable features included prominent Kupffer cells along sinusoidal linings and visible bile canaliculi in portal tracts (Fig. 12B1, B2).

**Fig. 13:**
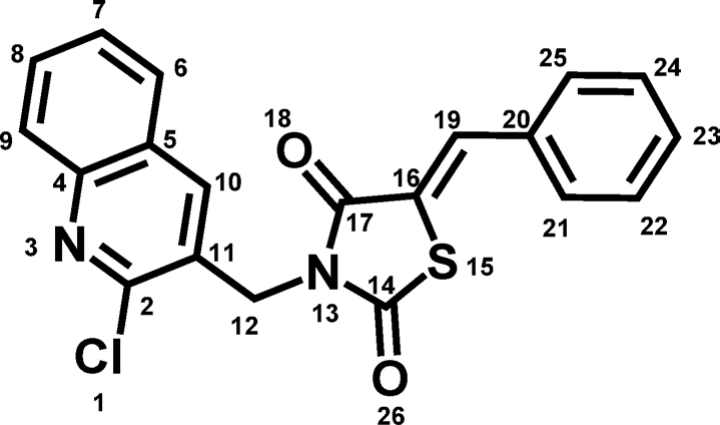
Chemical structure of compound **7** with its numbering by GLORYx.

It has been proposed that the oxidative cleavage of the TZD ring is a convinced metabolic pathway leading to the formation of reactive intermediates, and hence TZD toxicity. Metabolic studies confirmed this perception by the presence of *S*-oxidized metabolites of Troglitazone, PIO, and Rosiglitazone^28–30^ The proposed mechanism from these studies depicted by initial CYP450-mediated *S*-oxidation of TZD leading to formation of an unstable TZD sulfoxide. Then, the formed intermediate undergoes spontaneous cleavage to a reactive α-keto isocyanate intermediate. This isocyanate intermediate is more likely to covalently bind to hepatic proteins such as glutathione (GSH) and consequently cause hepatic failure^22^ In order to predict the metabolic positions on the TZD ring in **7**, it was submitted to GLORYx server, a reliable tool for forecasting the metabolites resulting from both phase I and phase II biotransformations^31^ The results provided the atom positions of **7** where metabolic reactions are most likely to initiate by CYP450, ranked by probability of occurrence from highest to lowest (Fig. 13; Table 1).

**Table 1:** The probability of metabolic biotransformation at each position of compound **7** proposed by GLORYx.

Atom	*P*	Atom	*P*	Atom	*P*	Atom	*P*	Atom	*P*
S15	0.584	C10	0.092	Cl	0.056	O26	0.016	C22	0.0
C23	0.165	C6	0.092	C14	0.052	C17	0.016	C20	0.0
C9	0.132	C19	0.088	C11	0.036	O18	0.004		
C12	0.12	N3	0.088	C2	0.036	C5	0.004		
C7	0.116	C16	0.056	C25	0.032	C4	0.004		
C8	0.104	N13	0.056	C21	0.032	C24	0.0		

“P” means probability.

These results indicated that the sulfur atom would be the primary site of metabolism, with a P of 0.584, while all other atom positions within the TZD exhibited significantly lower probabilities, each falling below 0.1. Other carbons within the quinoline and phenyl rings exhibited a substantially higher probability for metabolic reactions than the TZD moiety. This suggests that the TZD of compound **7** may be less susceptible to metabolic transformations, except sulfur atom.

**Fig. 14:**
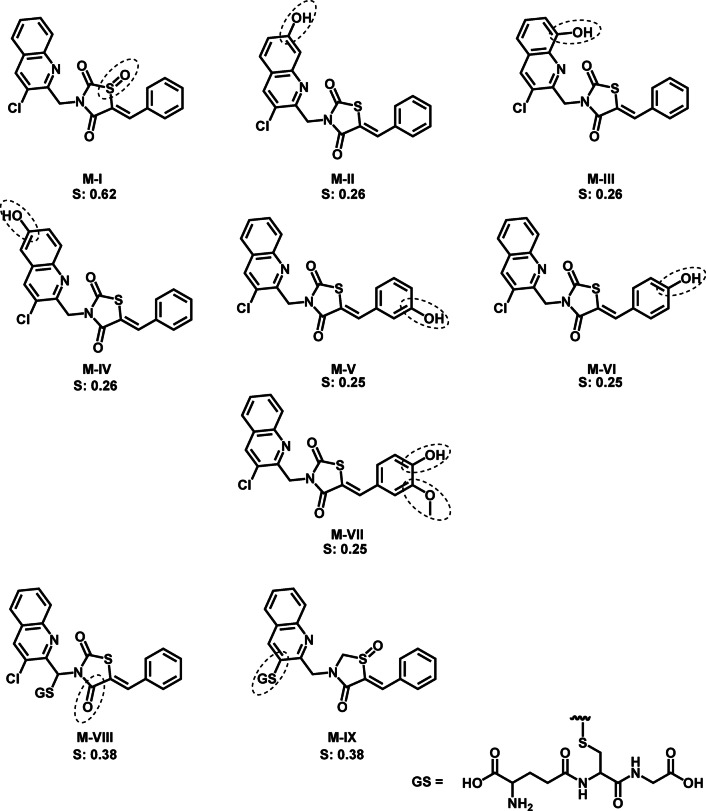
Predicted metabolic structures of compound **7** through phase I and II using GLORYx.

Furthermore, the investigation extended to the structural elucidation of the predicted metabolites using GLORYx server, depicted in Fig. 14. These predictions revealed that the foremost metabolic transformation involved the oxidation of the sulfur atom **M**-**I**, while there were no observed metabolites with a cleaved TZD ring. Subsequently, the second-highest probabilities were associated with hydroxylation reactions at C5, C7, and C8 of the quinoline **M**-**II**: **M**-**IV**, each possessing an equal likelihood of 0.26, followed by hydroxylation of phenyl group **M-V**: **M-VII** (P: 0.25). Interestingly, the analysis also observed the glutathionation process in phase II metabolism *via* conjugation at the methine group **M**-**VIII**, which served as a Michael acceptor, along with C2 of the quinoline moiety **M**-**IX**. These results indicated the high possibility of metabolic reactions to locate at sulfur atom through *S*-oxidation without ring cleavage. This observation may be attributed to the substitution at the nitrogen atom of the TZD moiety, which likely prevents hydrolysis of TZD and consequently the formation of reactive metabolites that could potentially induce toxicity^20^ These findings reveal that **7** could be less toxic than the current glitazones.

#### Morphological changes in pancreatic tissue

**Fig. 15:**
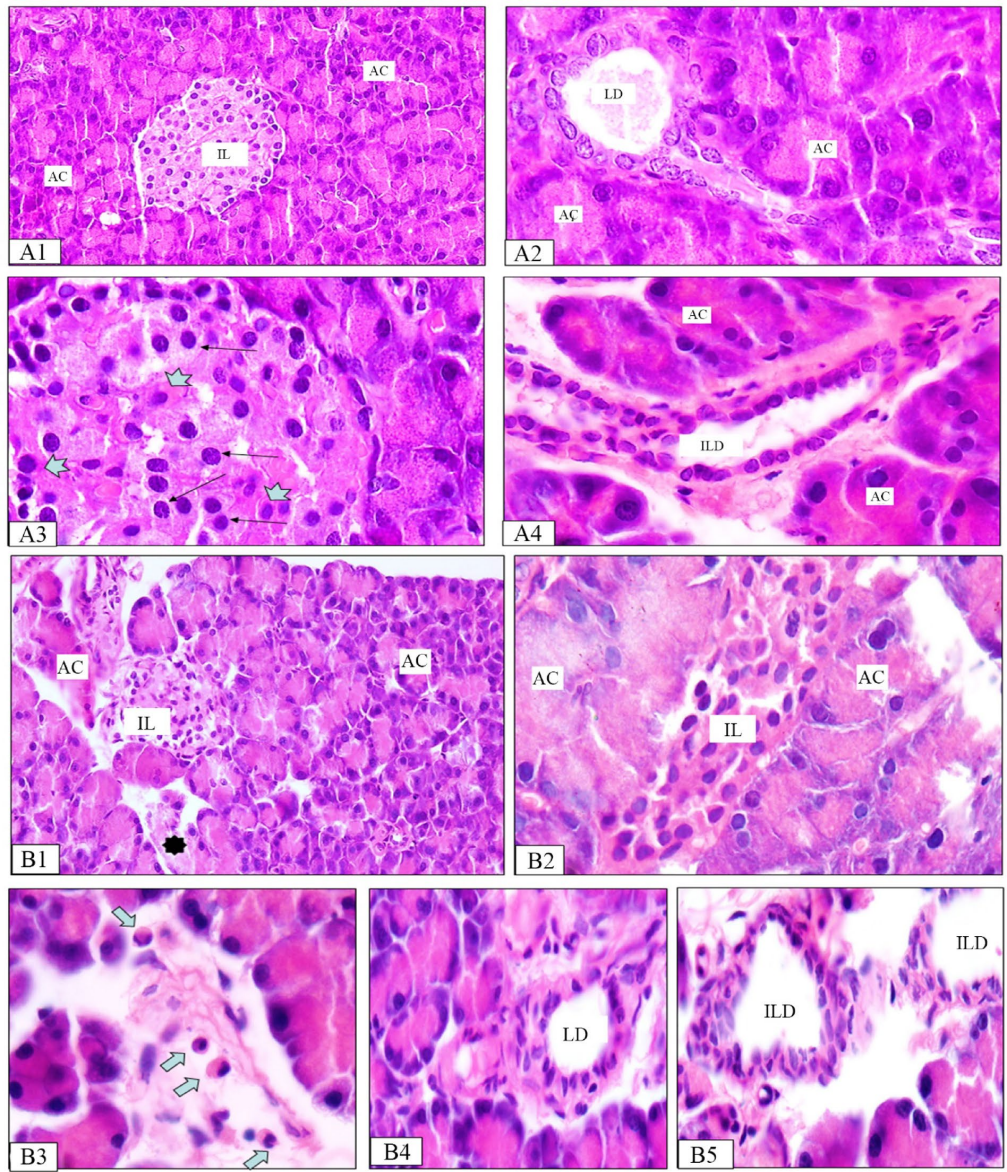
Photomicrograph of rat pancreatic tissue obtained from normal and diabetic groups and stained with hematoxylin and eosin (H & E). (**A1-A4**) Control group showing pancreatic acini with its basal basophilia and apical acidophilia (AC), Islets of Langerhans (IL) containing cells that form cords separated by a network of blood capillaries. α-Cells at the periphery (thick arrows) and β-cells at the center (thin arrows). (**B1-B3**) Diabetic group showing some islet’s cells with pyknotic and/or fragmented nuclei. The degenerated cells surrounded by empty spaces were filled with amyloid-like material. Some islets appear completely devoid of cells (black star). (**B4-B5**) Intralobular and interlobular ducts showed stratification of their epithelial lining (LD & ILD).

Histopathological studies were performed to evaluate the effect of **7** and PIO on pancreatic islets. The normal histological structure of the pancreatic tissue appeared in the form of lobules packed with acini that were separated from each other by very little connective tissue septa (Fig. 15A1-A4). It was reported that alloxan causes degeneration and necrosis of pancreatic β-cells^32,33^ The results showed that diabetic pancreatic tissue was characterized by marked morphological changes in the form of widening of the interlobular connective tissue containing numerous congested blood vessels loaded with RBCs (Fig. 15B1-B5). The inflammatory cells, mainly neutrophils, and eosinophils, were also seen surrounding ducts.

**Fig. 16:**
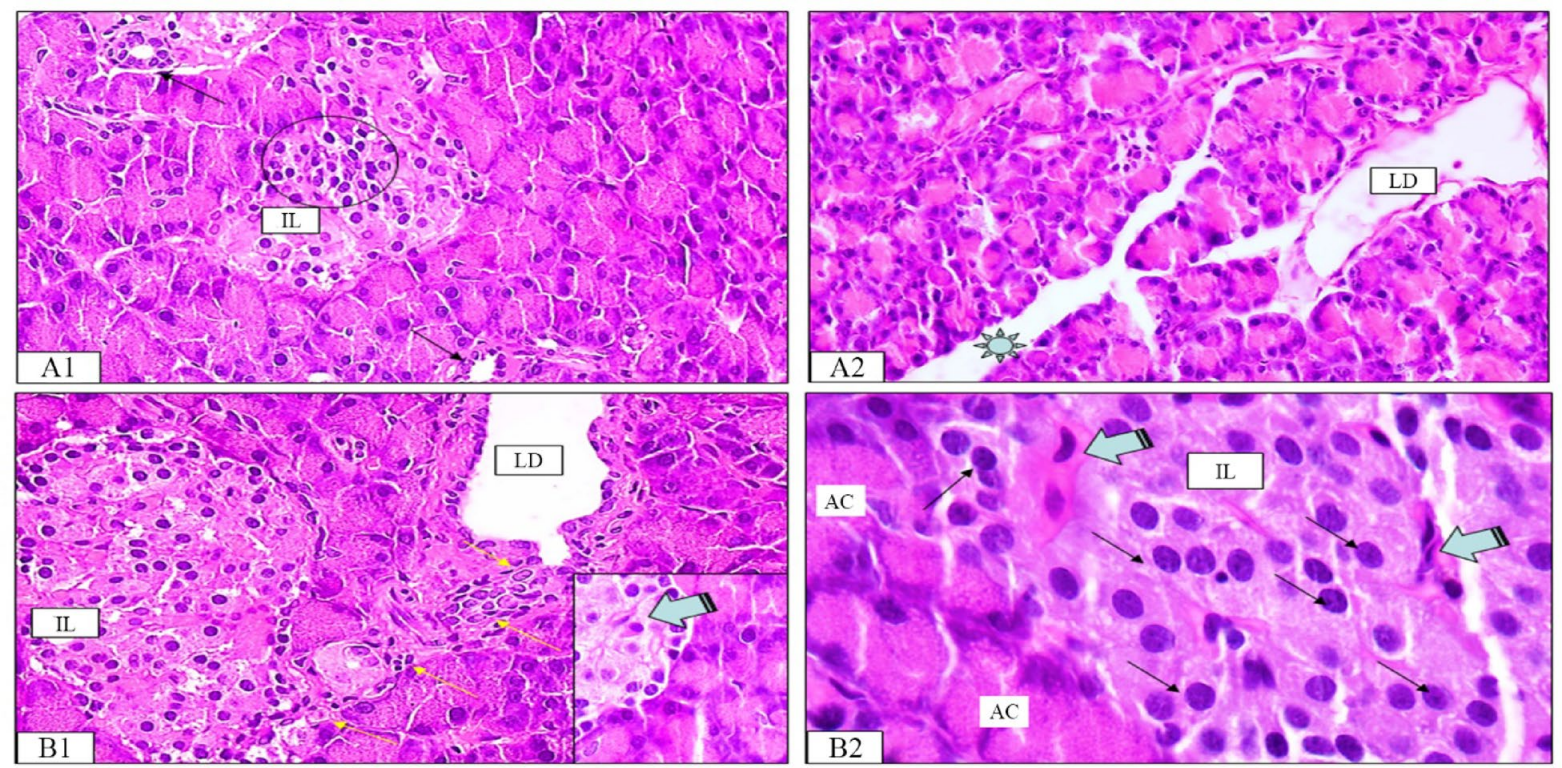
Photomicrograph of rat pancreatic tissue obtained from PIO- and **7**-treated groups and stained with H&E. (**A1-A2**) PIO-treated group showing scattered areas of small lobules separated by abundant connective tissue. Many islets showed an increase in cellular density, reduction of the inflammatory cellular infiltration, and more numerous β-cells occupying the center of the islets (empty circle), LDs were dilated and lined with flat cells and filled with accumulated secretion. (**B1-B2**) **7**-treated group showing normal pancreatic tissue. The islet cells appeared normal with fibroblast-like cells infiltrating the islets (stripped arrows). An islet showing connection by a stream of cells to the nearby duct that showed nearly normal lining epithelium (yellow arrows).

However, the pancreatic tissue of diabetic group treated with PIO showed signs of improvement in tissue organization (Fig. 16A1-A2). The pancreatic lobules appeared more organized with intervening connective tissue. The remaining islets demonstrated better preservation with reduced inflammatory cellular infiltration compared to the diabetic control group. These results agree with studies that demonstrated the protective effects on pancreatic β-cells exerted by PIO^34^ The pancreatic tissue of **7**-treated group showed marked improvement which was observed in the morphological features (Fig. 16B1-B2). The overall tissue architecture appeared more preserved compared to the diabetic control group, except some blood vessels remained congested, and certain ducts showed dilation with retained secretions. The reduction in inflammatory infiltration and better preservation of tissue architecture suggest that compound **7** may exert protective effects on pancreatic tissue similar to PIO, possibly through anti-inflammatory and antioxidant mechanisms. These protective effects could help maintain pancreatic function during the diabetic state, though longer-term studies would be needed to evaluate any potential effects on tissue regeneration.

### Molecular docking study

Molecular docking studies were performed to investigate the binding mode of compound **7** within the PPARγ LBD, and to compare it with the reference drug PIO. The PPARγ LBD has a large Y-shaped ligand binding pocket. PIO adopts a U-shaped conformation within the binding site with the hydrophobic chains wrapping around H3. TZD of PIO is reported to exist adjacent to H12 where the nitrogen atom makes a hydrogen bond with the hydroxyl group of Tyr473 in H12, stabilizing the active conformation of H12. Additionally, two carbonyl groups of the TZD head group make hydrogen bonds with the side chains of His323, Ser289, and His449. The phenyl group of the PIO engaged in hydrophobic interaction with amino acid Cys285^35^.

Docking studies of compound **7** on PPARγ, complexed with PIO as a co-crystallized ligand (PDB code: 5Y2O), were performed using AutoDock software. To validate the docking protocol, PIO was re-docked onto the binding domain of PPARγ. PIO docked at almost the same position with a binding score of -9.62 kcal/mol with RMSD equals 1.73 Å.

**Fig. 17:**
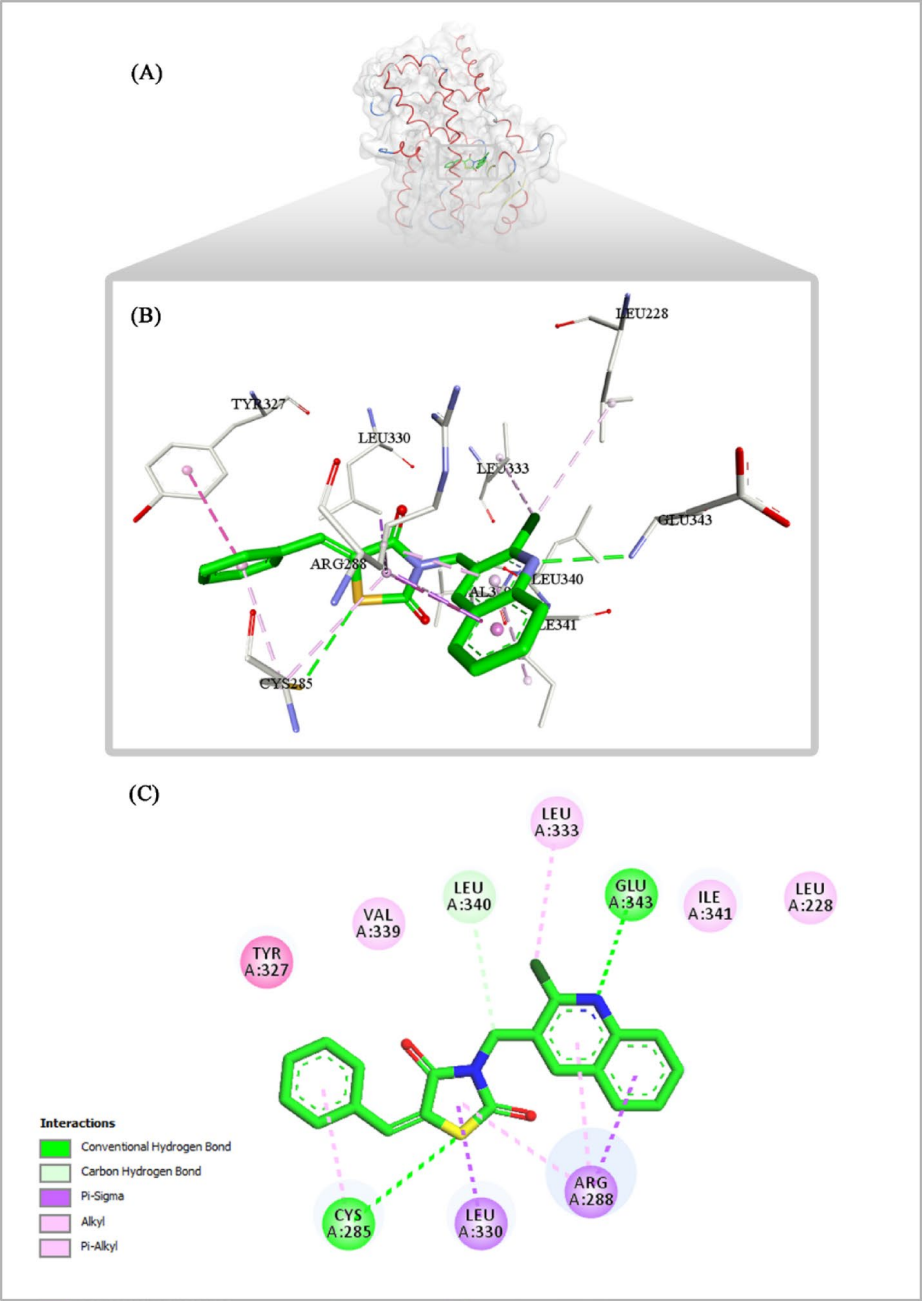
(**A**) Overall structure of the PPARγ LBD complexed with compound **7** (green); (**B**) 3D and (**C**) 2D binding mode of **7** with PPARγ LBD.

The docking results also revealed a high binding score for **7** (-9.66 Kcal/mol). The analysis revealed that the binding pose of compound **7** typically lies between arm I and arm II of the PPARγ binding pocket (Fig. 17). The phenyl ring of compound **7** showed the same interactions with key amino acid Cys285 (arm I), similar to the co-crystallized ligand. The quinoline ring of compound **7** played a critical role in stabilizing the molecule through multiple interactions (arm II). The quinoline ring itself established a π-π stacking interaction with Arg288, while the nitrogen atom of the quinoline moiety formed a hydrogen bond with Glu343. Furthermore, the chloro substituent of the quinoline ring participated in hydrophobic interactions with Leu333, enhancing the compound’s affinity in this region of the binding pocket (Fig. 17). Finally, TZD ring formed a network of π-sigma interactions with Leu330 and Arg288, further reinforcing the compound’s positioning within the binding pocket.

### In silico ADME prediction

The ADME properties of compound **7** were evaluated using the SwissADME server and compared with PIO as a reference drug^36^ The results (Table S1) confirmed that compound **7** complies with Lipinski’s rule of five, with no violations, suggesting favorable oral bioavailability. The pharmacokinetic predictions indicate high gastrointestinal (GI) absorption, further supporting the compound’s suitability for oral administration. The blood-brain barrier (BBB) permeability is predicted to be low, suggesting that compound 7 is unlikely to cause central nervous system (CNS)-related side effects. Moreover, compound **7** is not a P-glycoprotein (P-gp) substrate, indicating it is not actively effluxed, which may contribute to improved bioavailability. Furthermore, compound **7** does not trigger any PAINS (Pan-Assay Interference Compounds) alerts. Additionally, the synthetic accessibility score of 3.29 suggests that compound **7** is relatively straightforward to synthesize, supporting its feasibility for synthesis.

**Fig. 18:**
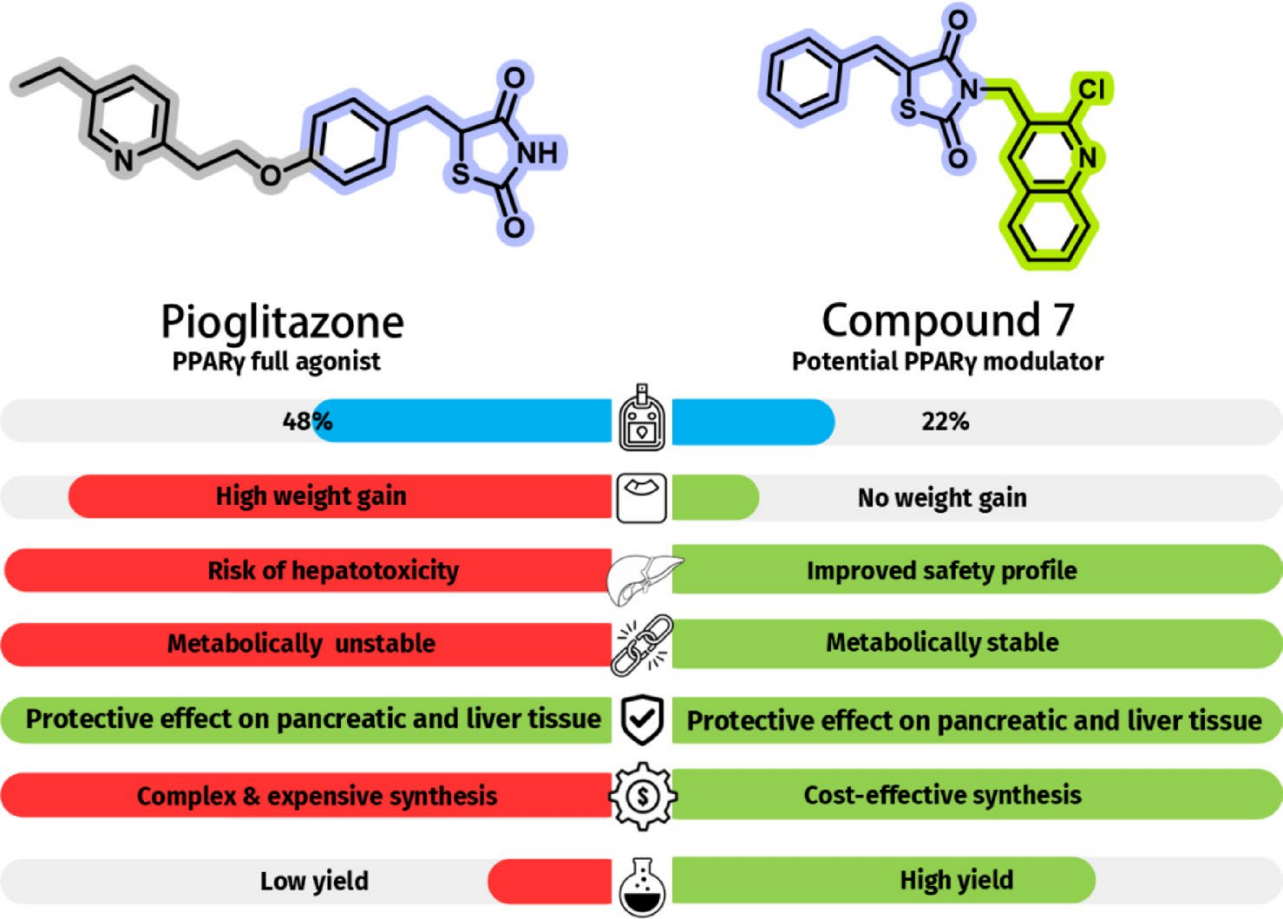
Comparative summary of biological and synthetic profiles of PIO and compound 7.

Overall, compound **7** shares the same biological profile and binding mode as SPPARMs, offering the benefits of partial receptor activation with reduced adverse effects (Fig. 18). Compound **7** represents a novel and safer alternative to PIO, providing a strong foundation for the future development of improved PPARγ modulators for diabetes treatment.

## Experimental

### Chemistry

#### Materials and methods

All required chemicals, solvents, and reagents were purchased from Sigma–Aldrich and Merck. Reaction progress was monitored using thin layer chromatography (TLC) on pre-coated Silica Gel Merck 60 F254 aluminum sheets, using hexane/ethyl acetate (2:8) as the mobile phase. TLC spots were visualized under UV light (254/365 nm). Melting points of the synthesized compounds were determined by open glass capillary tubes and are uncorrected. The^1^H NMR and^13^C NMR spectra were recorded on Bruker model DRX-500 and 100 MHz, respectively, in DMSO-*d*_6_ using tetramethylsilane (TMS) as the internal standard. Chemical shift values are given in δ (ppm) and the signals are described as s (singlet), d (doublet), t (triplet), q (quartet), and m (multiplet) whereas coupling constants (*J*) are expressed in Hz. Mass spectra were recorded on an Advion Compact Mass Spectrometer (CMS) using electrospray ionization (ESI) with amlodipine as standard. HPLC analysis was performed on an Agilent 1260 Infinity II system using 100% acetonitrile as the mobile phase.

#### Synthesis of 2-chloroquinoline-3-carbaldehyde 1

Yellow crystals, (4.1 gm) 42% yield, m.p.: 144–146 °C (lit. m.p.: 146–148 °C)^37^.

#### Synthesis of (2-chloroquinolin-3-yl)methanol 2

White powder, (0.35 gm) 91% yield, m.p.: 166–167 °C (lit. m.p.: 166–168 °C)^38^.

#### Synthesis of 2-chloro-3-(chloromethyl)quinoline 3

Light brown crystals, (0.293 gm) 92% yield; m.p.: 115–116 °C (lit. m.p.: 116–117 °C)^38^.

#### Synthesis of 1,**3-thiazolidine-2**,**4-dione 4**

White crystals, (5.69 gm) 81% yield, m.p.: 126–127 °C (lit. m.p.: 125–127 °C)^39^.

#### **Synthesis of (*Z*)-5-benzylidenethiazolidine-2**,**4-diones 5**

Pale yellow powder, (0.32 gm) 78% yield, m.p.: 237–238 °C (lit. m.p.: 237–238 °C)^40^.

#### Synthesis of (*Z*)-5-benzylidene-1,3-thiazolidine-2,4-dione potassium salt 6

White powder, (0.276 gm) 76% yield, m.p.: >300 °C.

#### Synthesis of (*Z*) 5-benzylidene-3-[(2-chloroquinolin-3-yl)methyl]thiazolidine-2,4-diones 7

Potassium salt of (*Z*)-5-benzylidenethiazolidine-2,4-dione **6** (1 mmol, 0.243 gm) was added to a stirred solution of intermediate **3** (1 mmol, 0.212 gm) in acetonitrile (50 mL) and heated under reflux. The progress of the reaction was monitored by TLC using 3:7 hexane : ethyl acetate as eluent. After the completion of the reaction, the solvent was evaporated under reduced pressure. Then, the residue was added to water, the formed precipitate was filtered off, washed with water, dried, and recrystallized from acetonitrile. White crystals, (0.239 gm) 63% yield, m.p.:235–236 °C; ^1^H NMR (500 MHz, DMSO-*d*_6_) δ 8.40 (s, 1 H, quinoline H4), 8.03 (d, *J* = 8.1 Hz, 1 H, quinoline H5), 7.97 (s, 1 H, methine C-H), 7.94 (d, *J* = 8.4 Hz, 1 H, quinoline H8), 7.79 (m, 1 H, quinoline H7), 7.64 (m, 3 H, phenyl 2 H and quinoline H6), 7.54 (m, 2 H, phenyl), 7.49 (m, 1 H, phenyl), 5.00 (s, 2 H, CH_2_); ^13^C NMR (100 MHz, DMSO-*d*_6_) δ 167.41, 165.50, 148.25, 146.28, 137.31, 133.38, 132.95, 130.96, 130.78, 130.19, 129.45, 127.99, 127.62, 127.51, 126.88, 126.50, 121.38, 42.60; HPLC analysis: Mobile phase: 100% MeCN, Retention Time (RT): 2.395 min, peak area: 94.89% at λ^max^ 236 nm; ESI-MS m/z: 380.5 [M + H]+ (calcd. for C_21_H_14_ClN_2_O_2_S, 380.86).

### Biological investigation

#### In vivo antidiabetic activity

In vivo antidiabetic study was determined by studying the effect of orally administered compound **7** on glucose tolerance on alloxan-induced non-insulin-dependent diabetes mellitus (NIDDM) in rats^33^ Twenty adult healthy male Albino Wistar rats (170–200 gm) were acquired from Deraya University, Minia, Egypt, and kept at room temperature with food and water *ad libitum*. Alloxan was freshly prepared in normal saline solution for inducing diabetes at a dose of 120 mg/kg body weight intraperitoneally to fifteen overnight-fasted rats, after 3 days of acclimatization. To overcome drug-induced hypoglycemia, the animals were permitted to drink only 5% glucose solution the whole night. The remaining five rats were injected with an equivalent volume of CMC as the normal control group (Group I). BGL was measured after 72 h for all rats using glucometer. Rats were considered diabetic when their BGL was found to be at least 250 mg/dL.^26^ Diabetic rats were then divided into three groups (five rats each). Diabetic control group (Group II) was only given the vehicle (0.25% CMC) orally. Diabetic rats were orally fed with PIO (Diabetin tablets, Unipharma^®^) as 0.25% CMC suspension at a dose of 36 mg/kg (Group III). Diabetic rats were orally fed with synthesized compound **7** (as 0.25% CMC suspension) at an equimolar dose (38 mg/kg) of the reference drug PIO (Group IV). BGLs were then measured at 0, 1, 7, and 15 days by collecting blood samples from the tail vein (caudal vein)^41^ The percentage of change in blood glucose level was calculated using the following formula^42^.

% lowering of blood glucose level = (C_F_ – C_L_) / C_F_ × 100.

C_F_ is the blood glucose concentration on day 0, and C_L_ is the blood glucose concentration after 15 days.

Overnight-fasted rats were weighed using an electric balance prior to the commencement of the study, and their weights were recorded on day 15 for body weight gain study.

After the end of the experiment, animals were euthanized using an overdose of Ketamine-Xylazine (300 mg/kg and 36 mg/kg, respectively) administered intraperitoneally. This method ensured that the animals were rendered unconscious and insensible to pain prior to sacrifice. Following confirmation of deep anesthesia through absence of pedal reflex and the induction of anesthesia, the animals were humanely sacrificed, and terminal blood collection was performed *via* cardiac puncture. Plasma samples were then collected for biochemical analysis. Pancreas was collected and divided into two portions. One portion for studying morphological changes in pancreatic tissue. The other portion of pancreas was freshly extracted with TRIzol™ reagent for total RNA. All animal experiments were approved by the Institutional Animal Ethics Committee (Approval No. 12/2023) at Deraya university. The study was conducted in accordance with the relevant guidelines and regulations, including National Institutes of Health (NIH) guidelines. Additionally, all procedures followed the ARRIVE (Animal Research: Reporting of In Vivo Experiments) guidelines to ensure proper reporting of in vivo studies.

#### PPARγ gene expression study

An ultrasonic homogenizer (Sonics-Vibracell, Sonics & Materials Inc., Newtown, USA) was used to homogenize 100 mg of pancreatic tissue in 1 mL of TRIzol™ solution (Amresco, Solon, USA). Total RNA was isolated from pancreatic tissues using the TRIzol™ RNA extraction reagent (Amresco, Solon, USA) according to the manufacturer’s instructions. The total RNA concentration was assessed at A_260_ nm, and the purity was computed using the A_260_/A_280_ ratio. When purity of the samples ≥ 1.7, samples were used for qRT-PCR utilizing GAPDH (Glyceraldehyde-3-phosphate dehydrogenase) as a reference housekeeping gene for determining the relative expression of PPARγ.

The RevertAid H Minus First Strand cDNA Synthesis kit (#K1632, Thermo Scientific Fermentas, St. Leon-Ro, Germany) was used to synthesize cDNA for equivalent amounts of total RNA in all samples, according to the manufacturer’s instructions. Single-stranded cDNAs were used in real-time PCR. SYBER Green [#K0251, Thermo Scientific Fermentas St. Leon-Ro, Germany-Maxima SYBER Green qPCR Master Mix (2X)] was used for PCR reactions and the StepOne Real-Time PCR Detection System (Applied Biosystems) was also used. Real-time polymerase chain reaction (qRT-PCR) was performed with 20 µL of RealMOD Green qRT-PCR Mix kit (iNtRON biotechnology) containing 0.02 µg RNA per reaction and 10 Pmol of specified primers for 30 cycles of 95 °C for 10 s. and 60 °C for 1 min. To assess the relative quantities of the products, the comparative threshold cycle (Ct) approach was utilized. The formula 2 ^(−ΔΔCt)^ was used to determine the relative expression. They were scaled in relation to controls, with control samples set to a value of one. The PPARγ primers used for rat-specific PPARγ were (forward: 5′- GCA TGG TGC CTT CGC TGA TG -3′; reverse: 5′- AGA ATA ATA AGG CGG GGA CGC − 3′) and GenBank accession number is NM_013124.3.

#### Hepatotoxicity studies

The collected samples of serum ALT and AST of the mice were quantified with ALT assay kits, according to the manufacturers’ protocols^43^.

Predicted metabolites of **7** were generated using the web-based GLORYx tool (https://nerdd.univie.ac.at/gloryx/*)*, University of Hamburg, Germany, simulating phase I and phase II metabolic reactions in humans using machine learning and site-of-reaction-based prediction models^31^ This was achieved by using the “phase I and II metabolism” option following the generation of SMILE notation using ChemDraw. Results from phase I and II metabolites with a corresponding score equal to or greater than 25% were selected.

#### Morphological changes in pancreatic tissue

One portion of pancreas was collected and stored in 10% neutral-buffered formalin, dehydrated in a graded ethanol series, cleared in xylene, and embedded in paraffin wax. Five Sects. (3–5 μm thickness) were stained with H&E and then examined under microscope at 400x and 1000x magnification^44^.

#### Statistical analysis

The data was encoded and entered using the Graph Pad Prism version 9 statistical package (Graph Pad, La Jolla, CA, USA). Statistical variations between groups were evaluated using one-way analysis of variance (ANOVA) followed by Bonferroni *t*-test. Results of all biological studies were expressed as means ± SD. P-values less than 0.05, was considered statistically significant, *p < 0.05.

### Molecular docking study

Molecular docking was performed using AutoDock. The crystal structure of PPARγ was obtained from RCSB Protein Data Bank (http://www.rcsb.org/*).* The crystal structure 5Y2O was selected for docking studies as it met the criteria of high-resolution data, well-defined binding sites, and their association with the specific ligand of interest in our research^45^ The input files for molecular docking were prepared using Discovery Studio (DS) 2016 client and AutoDock tools 4.2.6 bundled with MGL tools (version 1.5.7). Prior to docking, the protein structure was subjected to necessary cleaning procedures, which involved removing small molecular ligands, heteroatoms, non-standard residues, and water molecules. The 3D structures of ligands were retrieved from ChemDraw as a single file in 3D-spatial data file (SDF) format. The structures of ligands were energy-minimized using a universal force field and saved in PDB format. Then, these files were imported to Autodock, the Gasteiger charges and polar hydrogens were added, and the ligands were set up for the rotatable bond. The prepared protein and ligand files were then converted into PDBQT format, which serves as an input file for AutoDock for molecular docking. The binding site of the ligand was chosen according to a literature survey and selected as the active grid center. The dimensions of grid box were chosen to include all atoms of the ligands, default parameters were utilized with a grid box (74 × 60 × 82) Å which centered at (-47.217 × -1.288 × 77.334) Å with 0.375 Å of grid spacing. Additionally, the docking process was validated using co-crystallized ligand to ensure its quality and reliability for subsequent docking studies and the RMSD was calculated using LigRMSD server (https://ligrmsd.appsbio.utalca.cl/). The molecular docking was proceeded using tested ligand and the protein-ligand conformation with the lowest binding energy was chosen and visualized by DS 2016 client.

### In silico ADME prediction

The ADME properties of compound **7** were predicted using the SwissADME online tool (http://swissadme.ch/)^36^. Molecular descriptors, pharmacokinetics, lipophilicity, and water solubility were analyzed to assess oral drug-likeness.

## Conclusion

In this study, we successfully designed, synthesized, and evaluated compound **7**, a novel quinoline-thiazolidinedione hybrid, as a potential SPPARM with improved safety and therapeutic potential for diabetes management. Compound **7** demonstrated effective antidiabetic activity, effectively lowering blood glucose levels while exhibiting a safer metabolic and histopathological profile compared to PIO. Notably, compound **7** showed lower hepatotoxicity, minimal weight gain, and enhanced metabolic stability, addressing key safety concerns associated with traditional thiazolidinediones. Furthermore, its protective effects on liver and pancreatic tissues highlight its potential as a safer therapeutic option.

Molecular docking studies confirmed the strong binding affinity of compound **7** to PPARγ, interacting with key amino acid residues within the PPARγ LBD. These findings support its role as a potential selective PPARγ modulator, capable of dissociating insulin-sensitizing effects from adverse side effects. Additionally, ADME predictions indicated that compound **7** complies with Lipinski’s rule of five, with no violations, suggesting favorable oral bioavailability and high gastrointestinal GI absorption, making it suitable for oral administration.

While our findings demonstrate the promising potential of compound **7**, certain limitations should be acknowledged. Direct PPARγ binding assays are essential for measuring its efficacy and comparing it with other partial agonists. Moreover, while computational ADME predictions provide valuable insights into compound **7**’s pharmacokinetic profile, further experimental pharmacokinetic studies are important to validate these findings and fully characterize ADME properties. These studies are planned as part of our future work to further explore its pharmacological potential.

## Electronic supplementary material

Below is the link to the electronic supplementary material.

Supplementary Material 1

## Data Availability

The datasets generated and analyzed during the current study are available from the corresponding author upon reasonable request. All relevant data supporting the findings of this study, including synthetic procedures, in vivo experimental results, NMR spectra, and HPLC chromatograms are included in the manuscript and its supplementary information files. Additional raw data such as molecular docking files, can be provided by the corresponding author upon request.

## Abbreviations

AC: Apical acidophilia
AF2: Activation function 2
ALT: Alanine transaminase
AST: Aspartate transaminase
BBB: Blood brain barrier
BC: Bile canaliculi
BGL: Blood glucose level
CMC: Carboxy methyl cellulose
CNS: Central nervous system
Ct: Threshold cycle
CV: Central veins
CYP: Cytochrome
CYP450: Cytochrome P450
DS: Discovery Studio
ESI: Electrospray ionization
GAPDH: Glyceraldehyde-3-phosphate dehydrogenase
GI: Gastrointestinal
GSH: Glutathione
H: Helix
H&E: Hematoxylin and eosin
IL: Islets of Langerhans
ILD: Interlobular ducts
LBD: Ligand-binding domain
LD: Intralobular ducts
NASH: Nonalcoholic steatohepatitis
NIDDM: Non-insulin-dependent diabetes mellitus
NIH: National Institutes of Health
P: Probability
PA: Portal areas
PAINS: Pan-assay interference compounds
PCOS: Polycystic ovarian syndrome
P-gp: P-glycoprotein
PIO: Pioglitazone
PPARγ: Peroxisome proliferator-activated receptor γ
PPREs: PPAR response elements
qRT-PCR: Real-time polymerase chain reaction
RMSD: Root mean square deviation
RXR: Retinoid X receptor
SAR: Structure–activity relationship
SPPARMs: Selective PPARγ modulators
T2DM: Type II diabetes mellitus
TLC: Thin layer chromatography
TMS: Tetramethylsilane
TZD: Thiazolidinedione

## References

[1] DeFronzo, R. A. et al. Type 2 diabetes mellitus. *Nat. Rev. Dis. Primers*. **1**, 15019 (2015). 27189025 10.1038/nrdp.2015.19

[2] Yki-Järvinen, H. Thiazolidinediones. *N. Engl. J. Med.* **351**, 1106–1118 (2004). 15356308 10.1056/NEJMra041001

[3] Yau, H., Rivera, K., Lomonaco, R. & Cusi, K. The future of thiazolidinedione therapy in the management of type 2 diabetes mellitus. *Curr. Diab Rep.* **13**, 329–341 (2013). 23625197 10.1007/s11892-013-0378-8

[4] Kahn, S. E. et al. Glycemic durability of rosiglitazone, metformin, or glyburide monotherapy. *N Engl. J. Med.* **355**, 2427–2443 (2006). 17145742 10.1056/NEJMoa066224

[5] Soccio, R. E., Chen, E. R. & Lazar, M. A. Thiazolidinediones and the promise of insulin sensitization in type 2 diabetes. *Cell. Metab.* **20**, 573–591 (2014). 25242225 10.1016/j.cmet.2014.08.005 PMC4192012

[6] Bansal, G., Thanikachalam, P. V., Maurya, R. K., Chawla, P. & Ramamurthy, S. An overview on medicinal perspective of thiazolidine-2,4-dione: A remarkable scaffold in the treatment of type 2 diabetes. *J. Adv. Res.* **23**, 163–205 (2020). 32154036 10.1016/j.jare.2020.01.008 PMC7052407

[7] Ahmadian, M. et al. PPARγ signaling and metabolism: the good, the bad and the future. *Nat. Med.* **19**, 557–566 (2013). 23652116 10.1038/nm.3159 PMC3870016

[8] Rosen, E. D. & Spiegelman, B. M. PPARγ: A nuclear regulator of metabolism, differentiation, and cell growth. *J. Biol. Chem.* **276**, 37731–37734 (2001). 11459852 10.1074/jbc.R100034200

[9] Mangelsdorf, D. J. & Evans, R. M. The RXR heterodimers and orphan receptors. *Cell* **83**, 841–850 (1995). 8521508 10.1016/0092-8674(95)90200-7

[10] Schoonjans, K., Staels, B. & Auwerx, J. The peroxisome proliferator activated receptors (PPARS) and their effects on lipid metabolism and adipocyte differentiation. *Biochim. Biophys. Acta*. **1302**, 93–109 (1996). 8695669 10.1016/0005-2760(96)00066-5

[11] Saltiel, A. R. & Olefsky, J. M. Thiazolidinediones in the treatment of insulin resistance and type II diabetes. *Diabetes* **45**, 1661–1669 (1996). 8922349 10.2337/diab.45.12.1661

[12] Nolte, R. T. et al. Ligand binding and co-activator assembly of the peroxisome proliferator-activated receptor-gamma. *Nature* **395**, 137–143 (1998). 9744270 10.1038/25931

[13] Lincoff, A. M., Wolski, K., Nicholls, S. J. & Nissen, S. E. Pioglitazone and risk of cardiovascular events in patients with type 2 diabetes mellitus: a meta-analysis of randomized trials. *JAMA* **298**, 1180–1188 (2007). 17848652 10.1001/jama.298.10.1180

[14] Tang, H. et al. Pioglitazone and bladder cancer risk: a systematic review and meta-analysis. *Cancer Med.* **7**, 1070–1080 (2018). 29476615 10.1002/cam4.1354 PMC5911601

[15] Tuccori, M. et al. Pioglitazone use and risk of bladder cancer: population based cohort study. *BMJ*. **352** (2016). 10.1136/bmj.i1541 PMC4816602 27029385

[16] Gelman, L., Feige, J. N. & Desvergne, B. Molecular basis of selective PPARγ modulation for the treatment of type 2 diabetes. *Biochim. Et Biophys. Acta (BBA) - Mol. Cell. Biology Lipids*. **1771**, 1094–1107 (2007). 10.1016/j.bbalip.2007.03.004 17459763

[17] Higgins, L. S. & Depaoli, A. M. Selective peroxisome proliferator-activated receptor γ (PPARγ) modulation as a strategy for safer therapeutic PPARγ activation. *Am. J. Clin. Nutr.* **91**, 267S–270S (2010). 19906796 10.3945/ajcn.2009.28449E

[18] Motani, A. et al. INT131: a selective modulator of PPAR gamma. *J. Mol. Biol.* **386**, 1301–1311 (2009). 19452630 10.1016/j.jmb.2009.01.025

[19] Taxak, N., Parmar, V., Patel, D. S., Kotasthane, A. & Bharatam, P. V. S-oxidation of thiazolidinedione with hydrogen peroxide, peroxynitrous acid, and c4a-hydroperoxyflavin: A theoretical study. *J. Phys. Chem. A*. **115**, 891–898 (2011). 21214231 10.1021/jp109935k

[20] Campos, M. L. et al. New pioglitazone metabolites and absence of opened-ring metabolites in new N-substituted thiazolidinedione. *Drug Metab. Dispos.* **46**, 879–887 (2018). 29618574 10.1124/dmd.117.079012

[21] Mohamed, M. F. A. & Abuo-Rahma, G. E. D. A. Molecular targets and anticancer activity of quinoline–chalcone hybrids: literature review. *RSC Adv.* **10**, 31139–31155 (2020). 35520674 10.1039/d0ra05594h PMC9056499

[22] Ibrahim, A. M. et al. Chemistry and applications of functionalized 2,4-thiazolidinediones. *Eur. J. Org. Chem.* **26**, e202300184 (2023).

[23] Sohda, T. et al. Studies on antidiabetic agents. II. Synthesis of 5-[4-(1-methylcyclohexylmethoxy)-benzyl]thiazolidine-2,4-dione (ADD-3878) and its derivatives. *Chem. Pharm. Bull. (Tokyo)*. **30**, 3580–3600 (1982). 7160012 10.1248/cpb.30.3580

[24] Madivada, L. R. et al. An improved process for pioglitazone and its pharmaceutically acceptable salt. *Org. Process. Res. Dev.* **13**, 1190–1194 (2009).

[25] Nazreen, S. et al. Design, synthesis, in silico molecular docking and biological evaluation of novel oxadiazole based thiazolidine-2,4-diones bis-heterocycles as PPAR-γ agonists. *Eur. J. Med. Chem.* **87**, 175–185 (2014). 25255433 10.1016/j.ejmech.2014.09.010

[26] Naim, M. J. et al. Synthesis, docking, in vitro and in vivo antidiabetic activity of pyrazole-based 2,4-thiazolidinedione derivatives as PPAR-γ modulators. *Arch. Pharm. (Weinheim)*. **351**, e1700223 (2018). 29400412 10.1002/ardp.201700223

[27] Kroker, A. J. & Bruning, J. B. Review of the structural and dynamic mechanisms of PPARγ partial agonism. *PPAR Res*. **2015** (2015). 10.1155/2015/816856 PMC4578752 26435709

[28] Reddy, V. B. G. et al. Mechanistic studies on the metabolic scission of thiazolidinedione derivatives to acyclic thiols. *Chem. Res. Toxicol.* **18**, 880–888 (2005). 15892582 10.1021/tx0500373

[29] Shen, Z. et al. Identification of novel metabolites of pioglitazone in rat and dog. *Xenobiotica* **33**, 499–509 (2008). 10.1080/0049825031000085951 12746106

[30] Alvarez-Sánchez, R., Montavon, F., Hartung, T. & Pähler, A. Thiazolidinedione bioactivation: A comparison of the bioactivation potentials of troglitazone, rosiglitazone, and Pioglitazone using stable isotope-labeled analogues and liquid chromatography tandem mass spectrometry. *Chem. Res. Toxicol.* **19**, 1106–1116 (2006). 16918252 10.1021/tx050353h

[31] De Bruyn Kops, C., Šícho, M., Mazzolari, A. & Kirchmair, J. GLORYx: Prediction of the metabolites resulting from phase 1 and phase 2 biotransformations of xenobiotics. *Chem. Res. Toxicol.* **34**, 286–299 (2021). 32786543 10.1021/acs.chemrestox.0c00224 PMC7887798

[32] Ramadan, B. K., Schaalan, M. F. & Tolba, A. M. Hypoglycemic and pancreatic protective effects of Portulaca oleracea extract in alloxan induced diabetic rats. *BMC Complement. Altern. Med* **17**, (2017). 10.1186/s12906-016-1530-1 PMC5225634 28077129

[33] Ighodaro, O. M., Adeosun, A. M. & Akinloye, O. A. Alloxan-induced diabetes, a common model for evaluating the glycemic-control potential of therapeutic compounds and plants extracts in experimental studies. *Med. (B Aires)*. **53**, 365–374 (2017). 10.1016/j.medici.2018.02.001 29548636

[34] Kimura, T. et al. Protective effects of pioglitazone and/or liraglutide on pancreatic β-cells in Db/db mice: comparison of their effects between in an early and advanced stage of diabetes. *Mol. Cell. Endocrinol.* **400**, 78–89 (2015). 25463759 10.1016/j.mce.2014.11.018

[35] Gampe, R. T. et al. Asymmetry in the PPARgamma/RXRalpha crystal structure reveals the molecular basis of heterodimerization among nuclear receptors. *Mol. Cell.* **5**, 545–555 (2000). 10882139 10.1016/s1097-2765(00)80448-7

[36] Daina, A., Michielin, O. & Zoete, V. SwissADME: a free web tool to evaluate pharmacokinetics, drug-likeness and medicinal chemistry friendliness of small molecules. *Sci. Rep.* **7**(1), 1–13 (2017). 10.1038/srep42717 PMC5335600 28256516

[37] Salahuddin, Mazumder, A. & Shaharyar, M. Synthesis, antibacterial and anticancer evaluation of 5-substituted (1,3,4-oxadiazol-2-yl)quinoline. *Med. Chem. Res.* **24**, 2514–2528 (2015).

[38] Bokosi, F. R. B. et al. Design, synthesis and biological evaluation of mono- and bisquinoline methanamine derivatives as potential antiplasmodial agents. *Bioorg. Med. Chem. Lett.* **38**, 127855 (2021). 33609655 10.1016/j.bmcl.2021.127855

[39] Elkholy, N. et al. Discovery of 3-(2-aminoethyl)-thiazolidine-2,4-diones as a novel chemotype of sigma-1 receptor ligands. *Chem. Biol. Drug Des.* **100**, 25–40 (2022). 35353926 10.1111/cbdd.14047

[40] Meirelles, L. V. et al. Diverse 3-methylthio-4-substituted maleimides through a novel rearrangement reaction: synthesis and selective cell imaging. *J. Org. Chem.* **87**, 2809–2820 (2022). 35108004 10.1021/acs.joc.1c02714

[41] Togashi, Y. et al. Evaluation of the appropriateness of using glucometers for measuring the blood glucose levels in mice. *Sci. Rep.* **6**, 25465 (2016). 27151424 10.1038/srep25465 PMC4858715

[42] Candasamy, M., Murthy, T. E. G. K., Gubiyappa, K. S., Chellappan, D. K. & Gupta, G. Alteration of glucose lowering effect of glibenclamide on single and multiple treatments with fenofibrate in experimental rats and rabbit models. *J. Basic. Clin. Pharm.* **5**, 62 (2014). 25278668 10.4103/0976-0105.139728 PMC4160721

[43] Naim, M. J. et al. Synthesis, molecular docking and anti-diabetic evaluation of 2,4-thiazolidinedione based amide derivatives. *Bioorg. Chem.* **73**, 24–36 (2017). 28582649 10.1016/j.bioorg.2017.05.007

[44] Lambert, J. D. et al. Hepatotoxicity of high oral dose (-)-epigallocatechin-3-gallate in mice. *Food Chem. Toxicol.* **48**, 409 (2010). 19883714 10.1016/j.fct.2009.10.030 PMC2905152

[45] Lee, M. A., Tan, L., Yang, H., Im, Y. G. & Im, Y. J. Structures of PPARγ complexed with lobeglitazone and pioglitazone reveal key determinants for the recognition of antidiabetic drugs. *Sci. Rep.* **7**, 16837 (2017). 29203903 10.1038/s41598-017-17082-x PMC5715099

