# Supplementary material for: Design and synthesis of a novel quinoline thiazolidinedione hybrid as a potential antidiabetic PPARγ modulator
Source: Sci Rep. 2025 Jun 1;15:19207. doi: 10.1038/s41598-025-03387-9 (PMC12127462; doi:10.1038/s41598-025-03387-9)
Supplement: Supplementary file 1 — Supplementary Material 1 [file 41598_2025_3387_MOESM1_ESM.docx]

**Design and Synthesis of a Novel Quinoline Thiazolidinedione Hybrid as a Potential Antidiabetic PPARγ Modulator**

Ayman M. Ibrahim^[a]^, Mai E. Shoman^[b]^, Radwa Taher Mohie el-dien^[c]^, Entesar Ali Saber^[d]^, Mahmoud Abdelnaser^[e]^, Sherif A. Maher^[f]^, Alaa M. Hayallah*^[g,h]^_,_ Mahmoud Abdul-Aziz El-Rehany^[e]^_,_ and Gamal El-Din A. Abuo-Rahma*^[a, b]^

*^[a]^ Department of Pharmaceutical Chemistry, Faculty of Pharmacy, Deraya University, New Minia, 61111, Egypt.*

*^[b]^ Department of Medicinal Chemistry, Faculty of Pharmacy, Minia University, Minia, 61519, Egypt*

*^[c]^ Department of Pharmacognosy, Faculty of pharmacy, New Valley University, El-kharga City, Egypt.*

*^[d]^ Department of Medical science, Histology and Cell biology, Faculty of Pharmacy, Deraya University, New Minia, 61111, Egypt.*

*^[e]^ Department of Biochemistry, Faculty of Pharmacy, Deraya University, New Minia, 61111, Egypt.*

*^[f]^ Department of Biochemistry, Faculty of pharmacy, New Valley University, El-kharga City, Egypt.*

*^[g]^ Department of Pharmaceutical Organic Chemistry, Faculty of Pharmacy, Assiut University, Assiut, 71526, Egypt.*

*^[h]^ Pharmaceutical Chemistry Department, Faculty of Pharmacy, Sphinx University, New Assiut, Egypt.*

^*^Correspondence

****To whom correspondence should be addressed.***

Alaa M. Hayallah, Pharmaceutical Chemistry Department, Faculty of Pharmacy, Sphinx University, New Assiut, Egypt, Department of Pharmaceutical Organic Chemistry, Faculty of Pharmacy, Assiut University, Assiut, 71526, Egypt.

**Tel.:** +201017915080

**E-mail address**: [alaa_hayalah@yahoo.com](mailto:alaa_hayalah@yahoo.com)

Gamal El‐Din A. Abuo‐Rahma, Department of Pharmaceutical Chemistry, Faculty of Pharmacy, Deraya University, New Minia, Minia, Egypt, Department of Medicinal Chemistry, Faculty of Pharmacy, Minia University, Minia, 61519, Egypt.

**Tel.:** +201003069431

**E-mail address**: [gamal.aborahma@mu.edu.eg](mailto:gamal.aborahma@mu.edu.eg); [gamal.aborahama@deraya.edu.eg](mailto:gamal.aborahama@deraya.edu.eg)


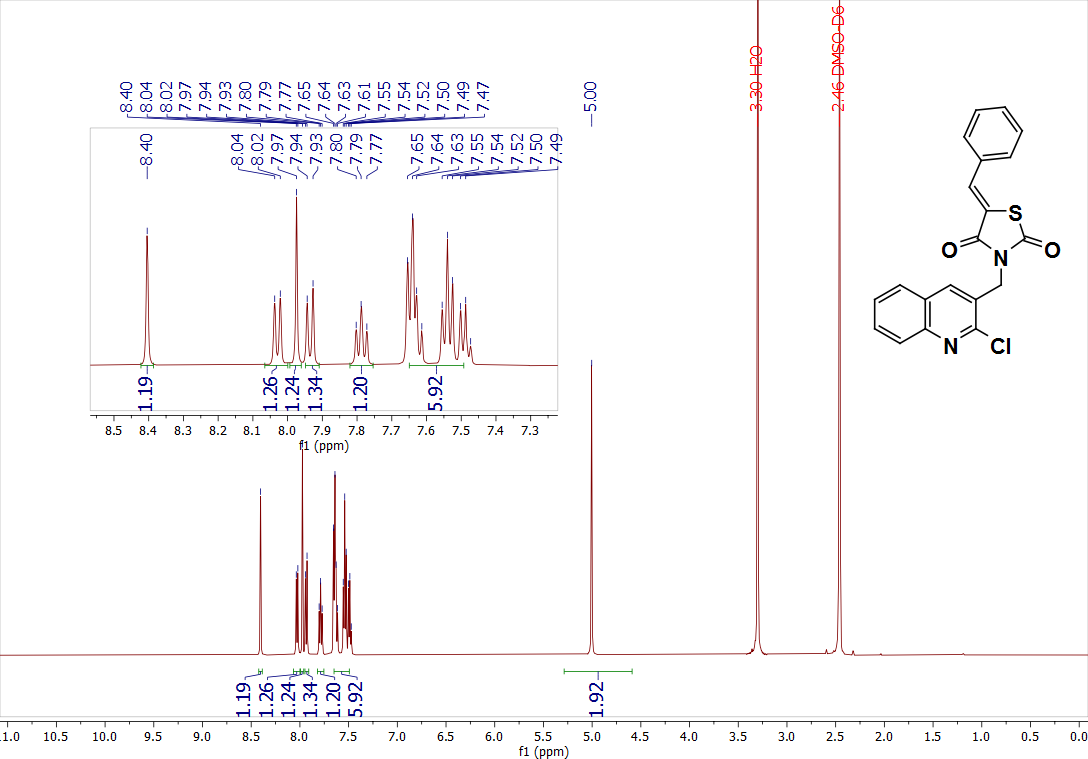


**Figure S1.** ^1^H NMR spectrum of compound **7** in DMSO-*d6* at 500 MHz.


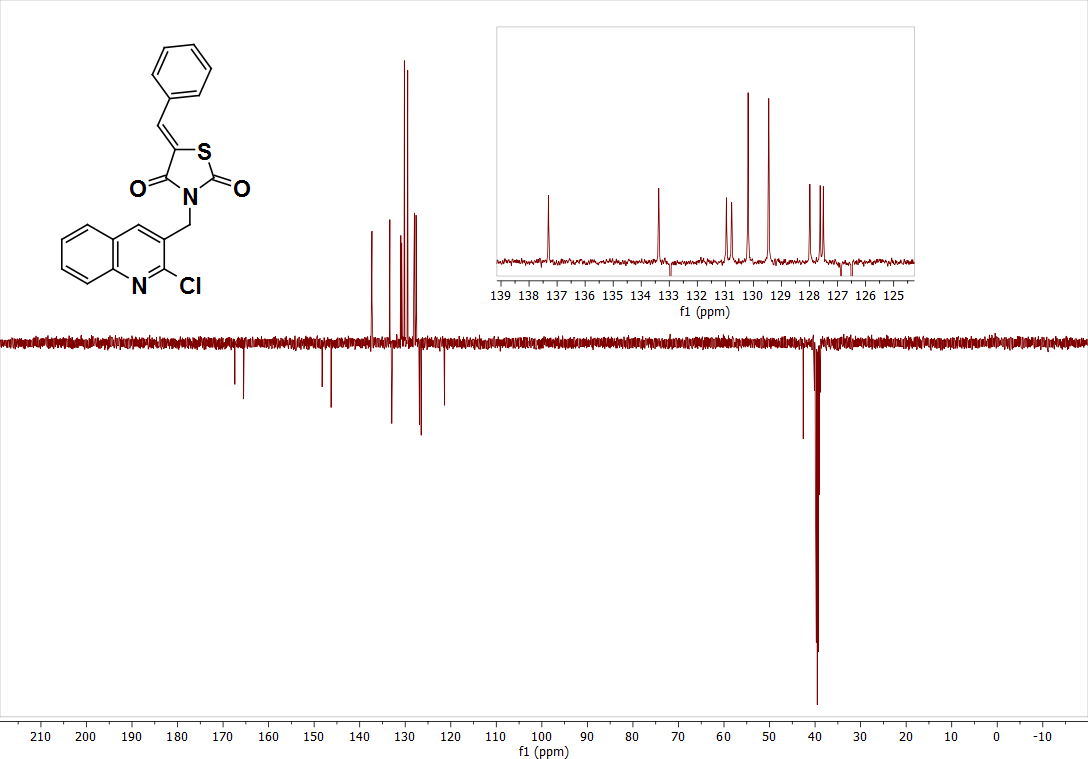


**Figure S2.** DEPTQ-135 ^13^C NMR spectrum of compound **7** in DMSO-*d6* at 100 MHz


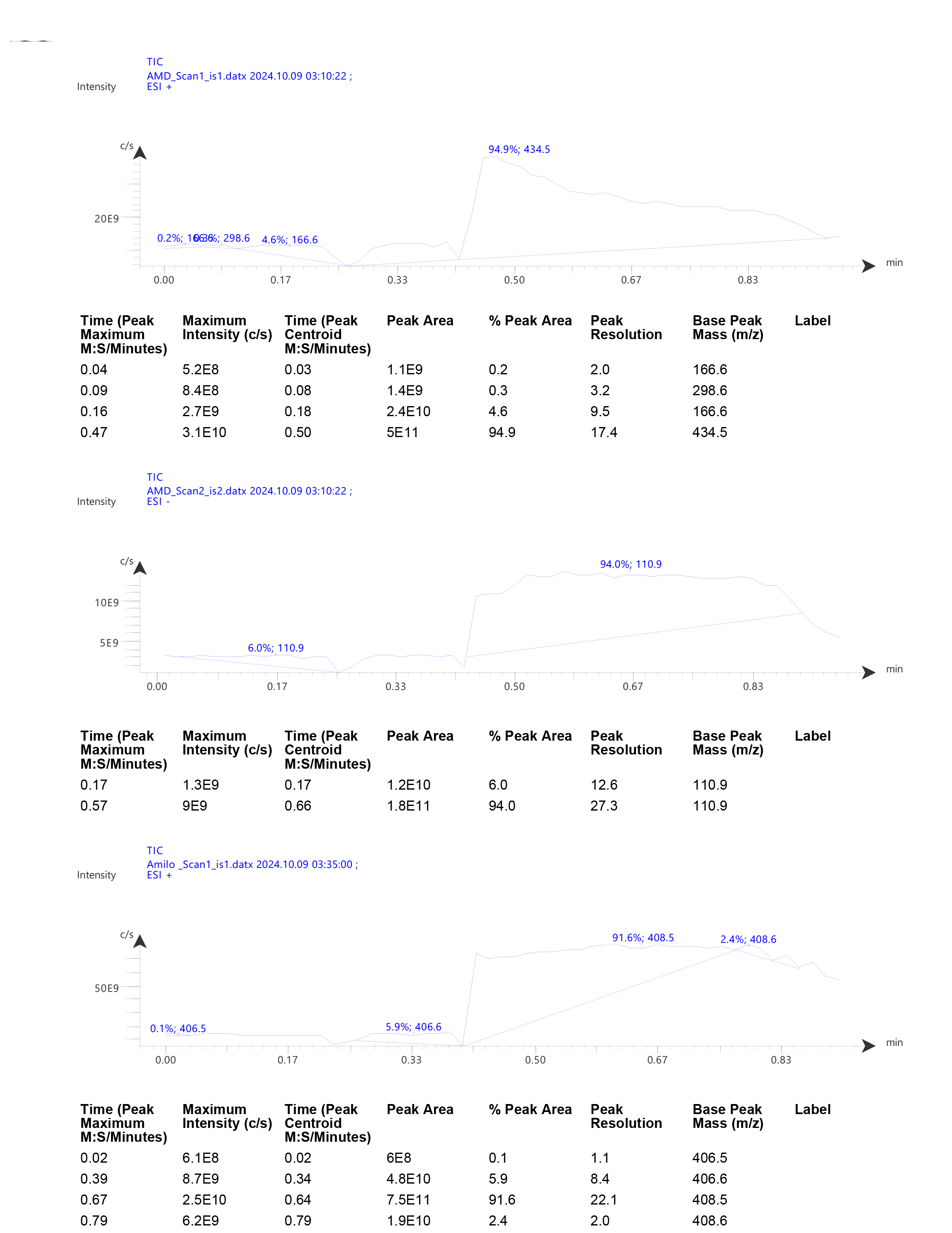


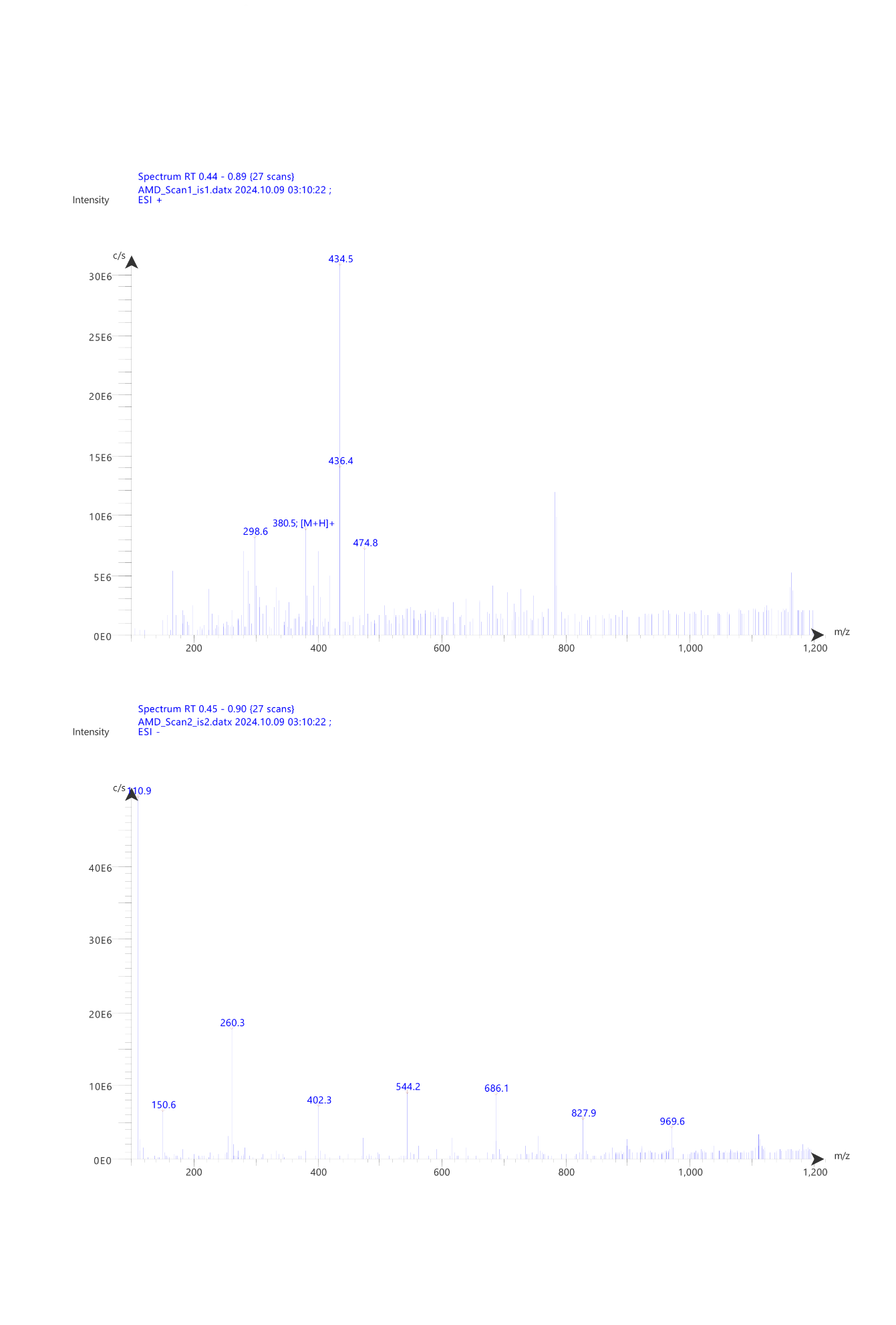


**Figure S3.** Mass spectrum analysis of compound **7**.


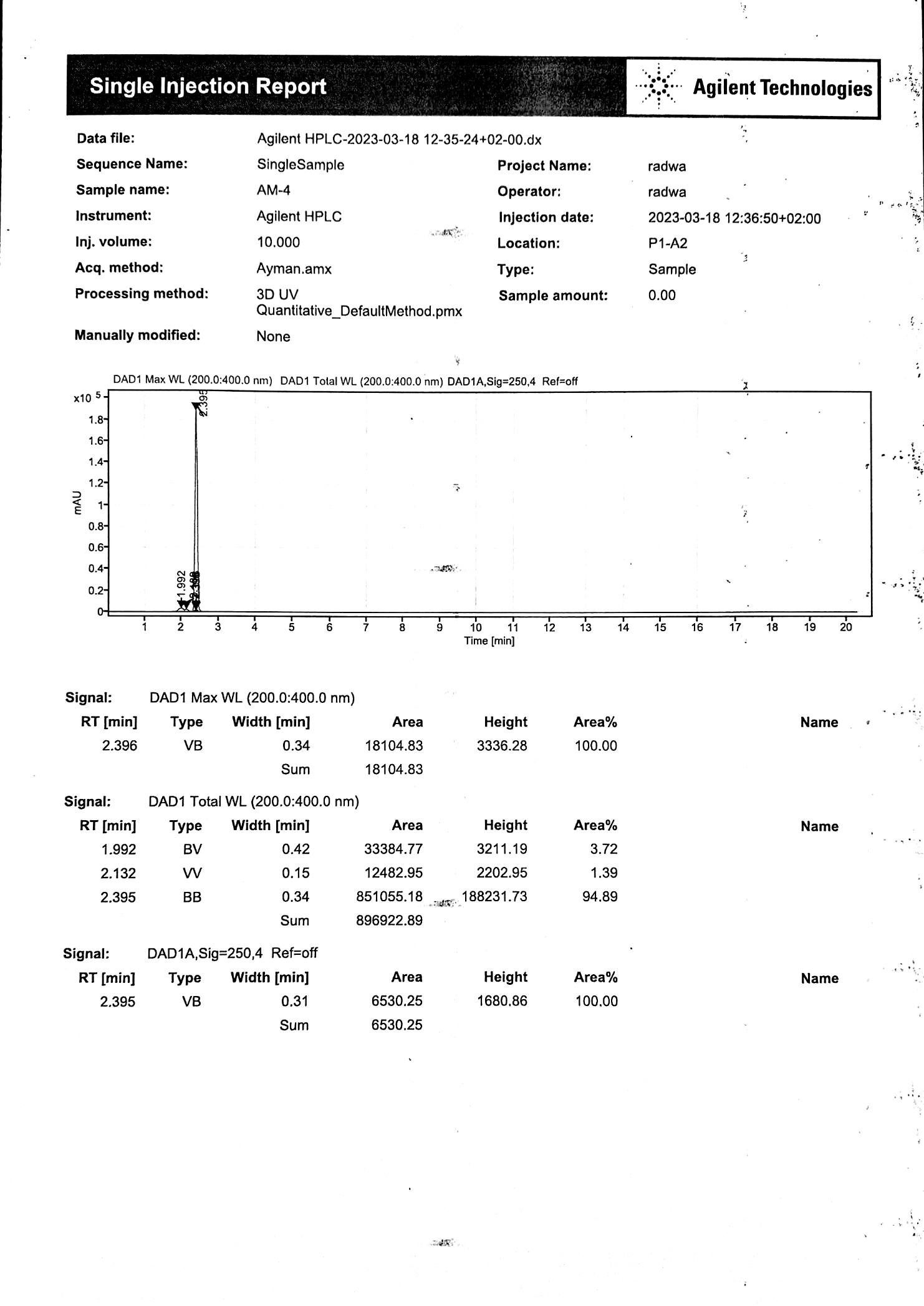


**Figure S4.** HPLC analysis of compound **7**.

**Table S1.** Predicted ADME properties of compound **7** compared to PIO, as evaluated using the SwissADME server.^1^

|  | Compound 7 | PIO |
| --- | --- | --- |
| Drug-likeness | | |
| Molecular weight (g/mol) | 380.85 | 356.44 |
| Water solubility | -5.67  Moderately soluble | -4.31  Moderately soluble |
| Hydrogen bond donor | 3 | 1 |
| Hydrogen Bond acceptor | 3 | 4 |
| Lipophilicity | 4.15 | 3.09 |
| Lipinski filter | Yes; 0 violation | Yes; 0 violation |
| Pharmacokinetics | | |
| GI absorption | High | High |
| Bioavailability Score | 0.55 | 0.55 |
| BBB permeant | No | No |
| P-gp substrate | No | No |
| CYP1A2 inhibitor | Yes | Yes |
| CYP2C19 inhibitor | Yes | Yes |
| CYP2C9 inhibitor | Yes | Yes |
| CYP2D6 inhibitor | No | Yes |
| CYP3A4 inhibitor | Yes | Yes |
| Log *K*_p_ (skin permeation) | -5.00 | -5.81 |
| Medicinal Chemistry | | |
| PAINS | 0 alert | 0 alert |
| Synthetic accessibility | 3.29 | 3.46 |

**References:**

1. Daina, A., Michielin, O. & Zoete, V. SwissADME: a free web tool to evaluate pharmacokinetics, drug-likeness and medicinal chemistry friendliness of small molecules. *Scientific Reports 2017 7:1* 7, 1–13 (2017).
